# The HANTS-fitted RSEI constructed in the vegetation growing season reveals the spatiotemporal patterns of ecological quality

**DOI:** 10.1038/s41598-024-65659-0

**Published:** 2024-06-26

**Authors:** Wenna Miao, Yue Chen, Weili Kou, Hongyan Lai, Ahmed Sazal, Jie Wang, Youliang Li, Jiangjie Hu, Yong Wu, Tianfu Zhao

**Affiliations:** 1 College of Forestry, Southwest Forestry University, Kunming, 650224 Yunnan China; 2 College of Big Data and Intelligent Engineering, Southwest Forestry University, Kunming, 650224 Yunnan China; 3 Kunming General Survey of Natural Resources Center, China Geological Survey, Kunming, 650111 China; 4 Yunnan Institute of Water Resources and Hydropower Research, Kunming, 650500 Yunnan China; 5 Lijiang Institute of Agricultural Sciences, Lijiang, 674100 Yunnan China; 6 Aerospace Science and Industry (Beijing) Space Information Application Co., Ltd, Kunming, 650111 China

**Keywords:** Ecological quality, RSEI, Land use change, Vegetation seasons, HANTS, Environmental impact, Ecological modelling

## Abstract

Yuxi, located in China’s central plateau of Yunnan, is grappling with ecological and environmental challenges as it continues to develop its economy. While ecological quality assessment serves as the foundation for ecological protection, it is pivotal to have reliable and long-term methods for assessing the ecological status to support informed decision-making in ecological protection. Reliable and long-term methods for assessing ecological status in order to facilitate informed decision-making in ecological protection are applied. This study utilized Landsat data to reconstruct four indices (greenness, wetness, dryness, and heat) during the vegetation growth in Yuxi from 2000 to 2020 that employs Harmonic Analysis of Time Series (HANTS) method. Subsequently, the annual Remote Sensing Ecological Index (RSEI) was computed by using the reconstructed indices to evaluate ecological quality in Yuxi. Additionally, spatiotemporal patterns and determinants of Yuxi’s ecological quality are unveiled through Sen’s slope estimator and Mann–Kendall test (Sen + MK) trend analysis, spatial auto-correlation analysis, and geographical detectors applied to year-by-year RSEI data. The findings in the paper indicate that the accuracy of the RSEI is significantly influenced by the vegetation season, suggesting that constructing the RSEI model with data from the vegetation growth season is crucial. Moreover, the HANTS optimization method effectively enhances the ecological indices used in the RSEI model, leading to smoother and more continuous filling of missing data. The difference between the reconstructed RSEI and the original RSEI falls within the range of − 0.15 to 0.15. Yuxi has an average RSEI of 0.54 to emphasis a moderate level of comprehensive ecological quality. Compared with river valley plains, the ecological quality of mountainous areas is higher, and the ecological quality of Yuxi presents a distinct center-edge pattern. From 2000 to 2020, Yuxi’s ecological quality exhibited fluctuations, with a slight overall improvement. Land use patterns, particularly in forestry land and impervious surfaces, are identified as the main drivers of these changes. The research offers valuable insights for scientific decision-making related to sustainable development and ecological protection.

## Introduction

The ecological environment is a complex system that involves the interaction of natural, social, and economic factors^1^. It plays a crucial role in human survival and sustainable social development, serving as a fundamental basis for human progress^2^. As population growth, economic advancement, and urban expansion continue, issues such as excessive resource consumption, soil erosion, desertification, and loss of biodiversity are becoming more pronounced. Maintaining a healthy ecological environment is essential for enhancing quality of life, strengthening the foundation for well-being and fostering social productivity^3^. Ecological quality serves as a key indicator of the overall health of the ecological environment, reflecting the impact of sustainable development strategies on the ecosystem. Assessing ecological quality is a valuable tool for evaluating the state of regional ecological environments, guiding the formulation of sustainable development plans and designing strategies for ecological protection^4^.

Remote sensing offers significant advantages in ecological quality assessment. Researchers can enhance the understanding of overall ecosystem condition through remote sensing with the coverage of large areas and comprehensive surface information. The data obtained through remote sensing platforms is acquired rapidly, which means it can reduce data acquisition time compared to traditional field survey methods and enable quick responses to ecological environment changes. Remote sensing technology can monitor surface changes by analyzing regular data, which provides reliable supports for long-term ecological quality assessment^5,6^. Remote sensing indices, such as the Normalized Difference Vegetation Index(NDVI)^7^, Leaf area index(LAI)^8^, and Soil adjusted vegetation index(SAVI)^9^, have been developed and utilized in ecological studies to effectively assess and quantify ecological quality dynamics^10,11^. The ecological environment is a complex system in which components are interconnected, and their interactions can impact the overall ecological quality. Therefore, relying on a single remote sensing indicator solely may not reflect regional ecological quality accurately^12^. It is essential to comprehensively evaluate ecological quality by integrating multiple indicators.

The Remote Sensing Ecological Index (RSEI) integrates into four factors (greenness, wetness, dryness, and heat) to create a comprehensive ecological index^13^ in the paper. Components of RSEI are not only strongly linked to ecological quality, but can also be derived exclusively from geospatial remote sensing technologies. Therefore, it can monitor the ecological quality objectively, low-costly, and quantitatively perform spatiotemporal visualization analysis^14^. Several studies utilized the RSEI model to monitor ecological quality alterations in interior cities^15^, coastal island cities^16,17^, and plateau basins^18,19^. These studies were carried out based on medium- and low-resolution satellite data. The MODIS satellite data products can control the quality availability to some extent through approaches including quality assurance and perspective limitations^20^. Nevertheless, the spatiotemporal consistency of satellite data products such as Landsat, SPOT, and Sentinel is inevitably still affected by unstable negative factors such as clouds, rain, climate, and problems with sensors^21^. Therefore, noise and other negative effects should be eliminated before applying the RSEI index. Local filtering and function fitting approaches have advantages in maintaining image details^22^. Various time series reconstruction techniques have been extensively elaborated, including Harmonic Analysis (HA), Asymmetric Gaussian (AG), Double Logistic (DL), Savitzky–Golay filter (SG), and Whittaker smoothing (WS). These techniques are valued for their simplicity in implementation and their capability to extract phenological markers from the time series^23^. Harmonic Analysis of Time Series (HANTS) is a method used for reconstructing time series data through harmonic analysis^24^. Studies have demonstrated that HANTS is capable of effectively filling in missing data while preserving the interannual variability present in the original dataset^25^. Moreover, the amplitude and phase of the harmonic components highlight the utility of HANTS in phenology research that can be utilized as quantitative metrics for evaluating vegetation phenology^26^.

Vegetation plays a crucial role in the ecosystem and is interconnected linked to ecological quality. The greenness in RSEI is influenced by the vegetation's growth status. Variations in vegetation information across different seasons can impact the representation of greenness data^27^. Selecting the appropriate time window for RSEI built is essential for ensuring the model's accuracy. However, existing RSEI research often ignores the condition of limiting image data in the vegetation growing season and introduces a large amount of data in non-vegetation growing seasons so that causes uncertainty in the results of RSEI inversion.

Yuxi is recognized as a prominent plateau of water town. The region's unique ecological characteristics are emphatic linked to its specific geography and climate. Any damage inflicted upon this ecosystem would be extremely challenging to reverse and could potentially result in irreversible ecological crises^28,29^. Yuxi has faced a various of challenges arising from socio-economic and environmental factors, such as eutrophication, debris flow disasters, severe landslides and drought conditions, which are endangered the ecological environment of the area. Thus, evaluating the ecological quality of Yuxi is crucial to the development of Yuxi. However, due to the climatic conditions in Yuxi, there is a significant difference between the vegetation growing season and the non-growing season. It is challenging to acquire high-quality remote sensing images during the rainy or growing seasons. The study aims to accomplish the following goals: (1) Explore the performance of RSEI in the vegetation growing season and non-growing season. (2) Verify the effectiveness of HANTS in constructing RSEI based on Landsat data. (3) Reveal the spatiotemporal spread as well as changing trends of Yuxi’s ecological quality. (4) Analyze the main factors affecting changes in Yuxi's ecological quality.

## Materials and methods

### Overview of the study region

**Figure 1:**
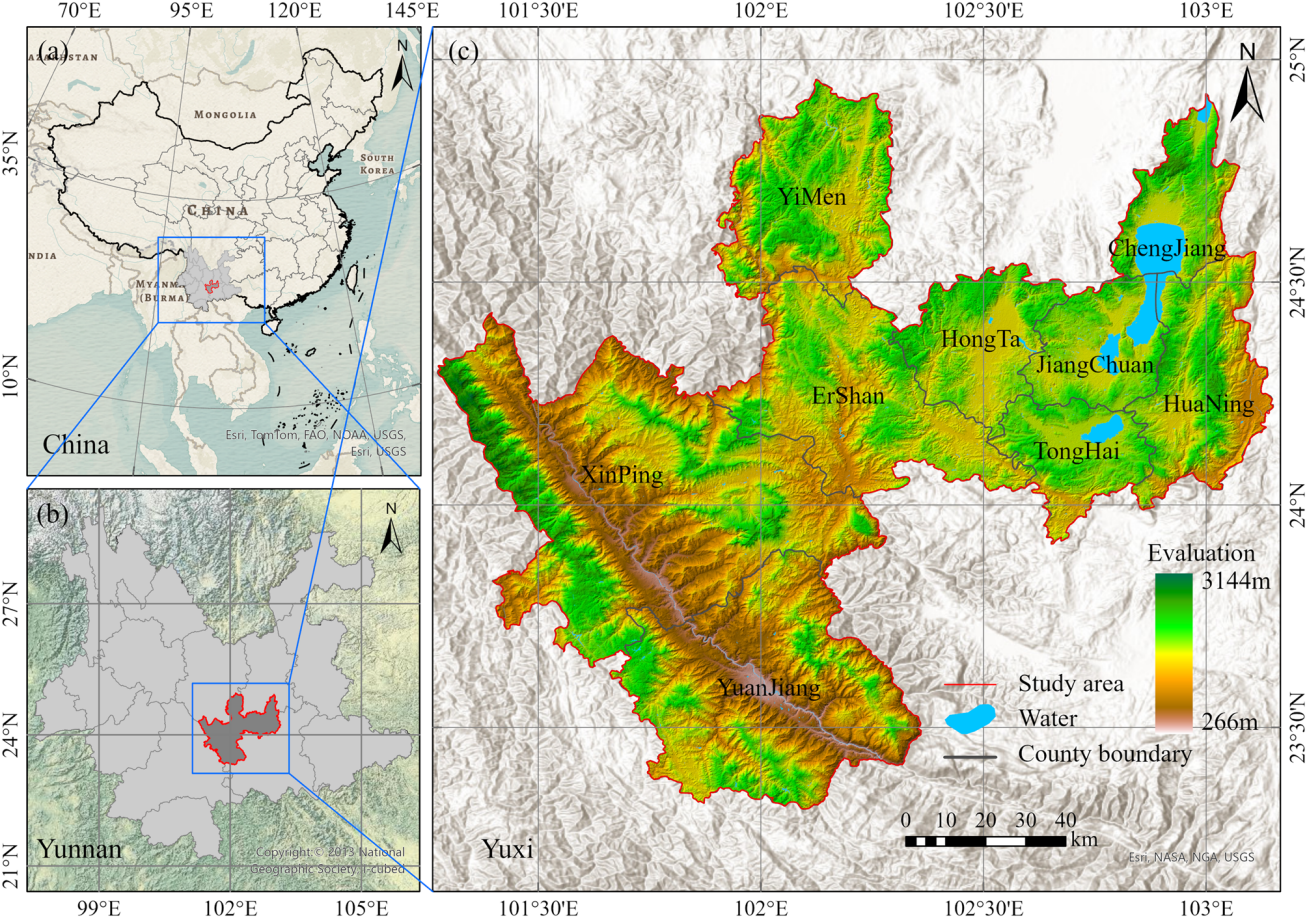
Geographical location and elevation map of the study region. The map is designed using ArcGIS Pro V2.5 software provided by the ESRI website (https://www.esri.com/en-us/arcgis/products/arcgis-pro/overview). The vector boundaries of the study area are sourced from the National Geoinformation Catalog Service (https://www.webmap.cn). The Digital Elevation Model (DEM) is from the ALOS DSM: Global 30 m v3.2 dataset provided by the JAXA Earth Observation Research Center (https://www.eorc.jaxa.jp/), accessed through Google Earth Engine (https://earthengine.google.com). (**a**) The base map is provided by ArcGIS Pro: Modern Antique (Esri, TomTom, Garmin, FAO, NOAA, USGS). (**b**) The base map is provided by ArcGIS Pro: USA_Topo_map (Copyright: ©2013 National Geographic Society, i-cubed). (**c**) The base map is provided by ArcGIS Pro: World_Hillshade (Esri, USGS).

The study area is focus on Yuxi, central plateau of Yunnan, spanning 23°30′–25° N latitude and 101°33′–103° E longitude. Yuxi covers a total area of 15,000 km^2^ and is located on the western edge of the Central Yunnan, in the transitional zone between the Ailao Mountains and the Hengduan Mountains, as shown in Fig. 1. The topography of Yuxi is marked by a diverse and complex terrain, with elevations ranging from 1500 to 3144 m. Three plateau lakes—Fuxian Lake, Xingyun Lake, and Qilu Lake—are found within its borders. The climate is characterized by humid and mild conditions, exhibiting a yearly mean temperature variation of 15.4–24.2 °C and a yearly precipitation range of 787.8–1000 mm^30^. The region encompasses both the arid season (November to April) and the annual wet season (May to October) that form a unique seasonal climate pattern^31^. With a forest coverage rate of approximately 64.06%, the area boasts rich forest resources^32^. The growth season of vegetation in Yuxi’s forest typically commences and concludes the rainy seasons, which coinciding with increased precipitation and higher temperatures^33,34^.

### Data and processing

#### Landsat series imagery

This study utilized Landsat series data (30 m resolution) from the Google Earth Engine (GEE) to reconstruct ecological indices time series and calculate RSEI. The Landsat series surface reflectance data (SR) available on GEE has undergone radiometric, geometric and atmospheric corrections, eliminating the need for preprocessing^35^. The data simply requires cropping to the study area, time filtering and cloud removal. During cloud removal, we first screened cloud cover less than 50% data in the research to ensure that the image covered the entire study area, then used GEE^36^. The algorithm is implemented in GEE through JavaScript programming. The Landsat data is presented in Table 1, with the research area identified as path/row 129-043. Over a 20-year period (2000–2020), Landsat 5 TM, Landsat 7 ETM+, and Landsat 8 OLI data were utilized. A total of 643 images from 2000 to 2011, 110 images from 2012 and 657 images from 2013 to 2020 were selected. Images from the vegetation growing season (May to October) were screened for median synthesis as the data source for model construction. It is important to note that GEE corrected the Landsat 7 ETM+ data using the SLC-off model. Visual inspection revealed that the 2012 image data in the study area contained complete information, consequently no further processing was conducted on the Landsat 7 ETM+ data in this article.

**Table 1:** Landsat dataset from 2000–2020.

Sensor	Time	Period	Images quantity	GEE datasets
Landsat 5 TM	2000–2011	May–October	640	LANDSAT/LT05/C02/T1_L2
Landsat 7 ETM+	2012	May–October	110	LANDSAT/LE07/C02/T1_L2
Landsat 8 OLI	2013–2020	May–October	657	LANDSAT/LC08/C02/T1_L2

#### Ancillary data

This study used various ancillary data sets, including Digital Elevation Models (DEM), temperature records, precipitation data, and land use type datasets (Table 2). DEM data are employed in creating an overview of the study region and determining terrain parameters such as slope, aspect and elevation. Land use type data were used to calculate the proportion of forest area, crops and impervious land in Yuxi from 2000 to 2020, and the factors affecting the ecological quality of Yuxi were jointly analyzed using temperature and precipitation data. All spatial data were projected using the WGS_1984_UTM_Zone_48N coordinate system and resampled to a consistent resolution of 30 m in the paper.

**Table 2:** Summary of auxiliary datasets used in the study.

Data	Products and source	Resolution	Pre-processing
Digital Elevation Model (DEM)	Advanced land observing satellite global digital surface mode (ALOS DSM): Global 30m v3.2 (https://www.eorc.jaxa.jp)	30 m	Calculate elevation, slope, and aspect
Temperature data	China 1 km resolution monthly average temperature dataset^37^	1000 m	Resample to 30 m and filter for the specified year
Precipitation data	China 1 km resolution monthly average precipitation dataset^37^	1000 m	Resample to 30 m and filter for the specified year
Land use type data	China land cover dataset (CLCD) dataset^38^	30 m	Calculate the proportion of land classes

### Method

**Figure 2:**
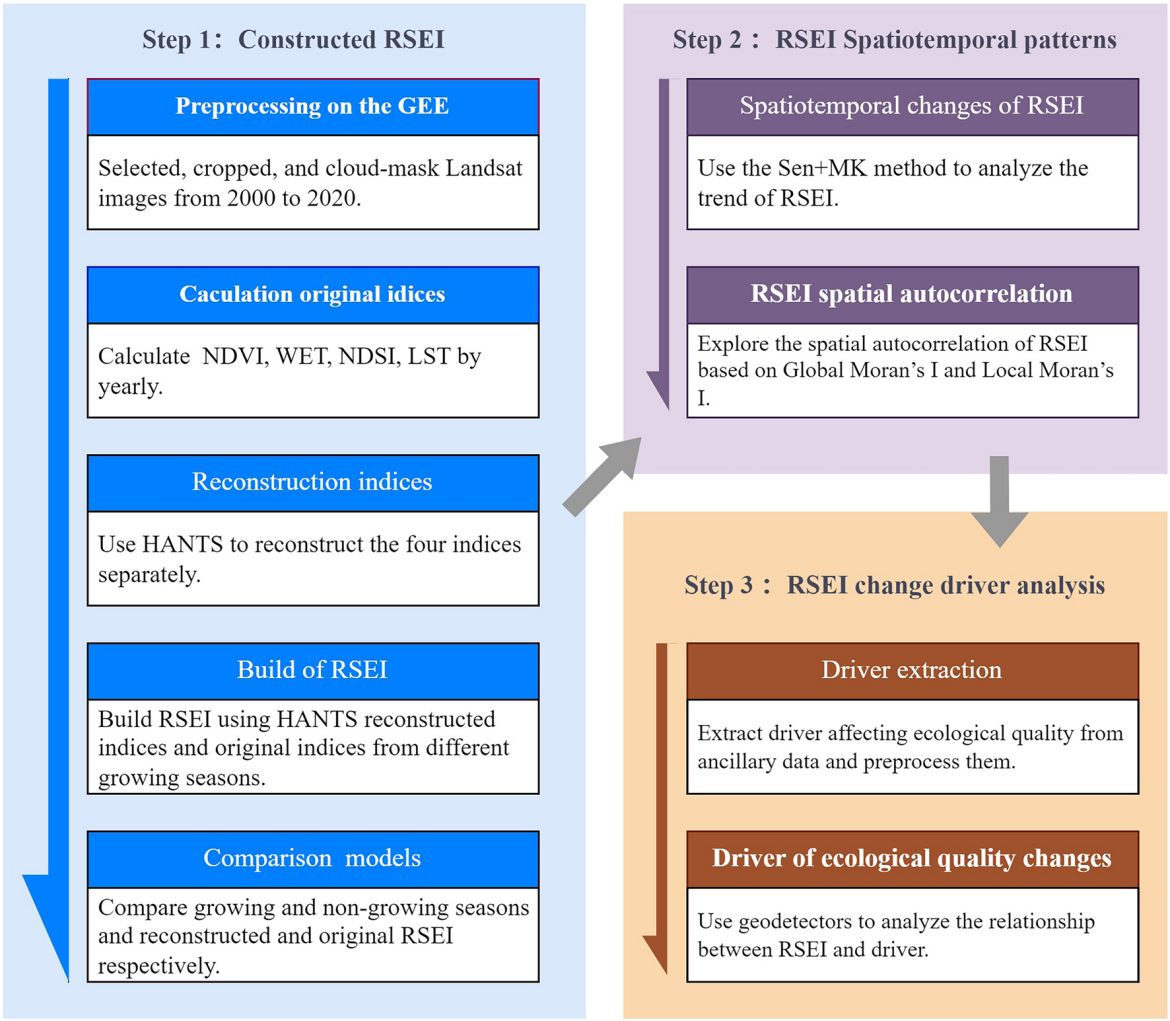
The study methodological workflow or framework.

The structured its workflow (Fig. 2) to meet the research aims, which essentially consist of three key parts: (1) Construction of the RSEI model and reconstruction of ecological indicator reconstruction. (2) Ecological quality assessment in the study region. (3) Analysis of spatiotemporal evolution of ecological quality in Yuxi and exploration of influencing factors.

#### RSEI component indices calculation

RSEI is a comprehensive evaluation model that combines four ecological parameters closely related to ecological quality: greenness, wetness, dryness and heat^13^. There are four ecological parameters that can represent RSEI:

1 $$\begin{array}{*{20}l} {RSEI = f\left( {Greenness,\;Wetness,\;Dryness,\;Heat} \right)} \\ \end{array}$$

where *Greenness*, *Wetness*, *Dryness*, and *Heat* include the normalized difference vegetation index (NDVI), tasseled cap Transform wetness index (TCW), Surface reflected temperature (LST), and Normalized difference bare soil index (NDBSI). Calculated from Eqs. (2)–(5):

2 $$\begin{array}{*{20}l} {NDVI = \frac{{\rho_{nir} - \rho_{red} }}{{\rho_{nir} + \rho_{red} }}} \\ \end{array}$$

3 $$TCW = c_{1} \rho_{blue} + c_{2} \rho_{green} + c_{3} \rho_{red} + c_{4} \rho_{nir} + c_{5} \rho_{swir1} + c_{6} \rho_{swir2}$$

4 $$NDBIS = \frac{SI + IBI}{2}$$

5 $$\begin{array}{*{20}l} {LST = \gamma \left[ {\varepsilon^{ - 1} \left( {\psi_{1} L_{sensor} + \psi_{2} } \right) + \psi_{3} } \right] + \delta } \\ \end{array}$$

where $\rho_{blue}$, $\rho_{green}$, $\rho_{Red}$, $\rho_{nir}$, $\rho_{swir1}$ and $\rho_{swir2}$ represent the B1, B2, B3, B4, B5, and B7 bands of the TM and ETM+ sensors, as well as the B2, B3, B4, B5, B6, and B7 bands of the OLI sensor. In Eq. (3), $c_{i}$ represents the tassel cap variation coefficient of TCW indices. The coefficients of Landsat TM, ETM+ and OLI are different^39–41^. Equation (4) calculated from soil index (SI) and building index (BI)^42^. Equation (5) represents a surface temperature inversion algorithm based on the single-channel method. In this algorithm, γ and δ are two parameters determined by the Planck function, $L_{sensor}$ representing radiance values. ε represents surface emissivity, while *ψ*_*1*_, *ψ*_*2*_, and *ψ*_*3*_ are three correction coefficients related to atmospheric water vapor content (w)^43^.

The NDVI, TCW and NDBSI are obtained through band calculation based on GEE. The LST data is used to the surface temperature product of the Landsat satellite. This product is obtained from the thermal infrared band through a dual-channel algorithm (Eq. 5).

#### Fitting indices based on HANTS

Harmonic Analysis of Time Series (HANTS) is a time series analysis method that combines both smoothing and filtering techniques. This method effectively utilizes the spatiotemporal characteristics of remote sensing images, linking spatial distribution patterns with temporal variations^24,44^. The core of this algorithm lies in Fourier transformation and linear regression using the method of least squares, where it decomposes time spectral data into multiple sine and cosine curves, including various frequencies. Afterwards, a subset of these curves that accurately represents the features of the time series is overlaid to accomplish the reconstruction of time series data^22,45^. The fundamental equations for HANTS data reconstruction are presented in Eqs. (6) and (7):

6 $$\begin{array}{*{20}l} {\tilde{y}(t_{j} ) = a_{0} + \mathop \sum \limits_{i = 1}^{nf} \left[ {a_{i} \cos \;(2\pi f_{i} ) + b_{i} \sin \;(2\pi f_{i} t)} \right]} \\ \end{array}$$

7 $$\begin{array}{*{20}l} {y(t_{j} ) = \tilde{y}(t_{j} ) + \varepsilon (t_{j} )} \\ \end{array}$$

where *y* denotes the original *NDVI*, *TCW*, *NDBSI*, or *LST* data; $\tilde{y}$ denotes the rebuilt data; and *ε* denotes the error sequence. $t_{j}$ denotes the time at which the observation *y* is made, where *j* ranges from 1 to *N*, and *N* represents the maximum length of the observation sequence. Where $nf$ signifies the count of periodic components in the time series, while *n* stands for the quantity of harmonics. The coefficient $a_{0}$ corresponds to the zero-frequency coefficient, which represents the average of the entire time series. $a_{i}$ and $b_{i}$ are the trigonometric components of frequency $f_{i}$. In this study, HANTS is implemented in the GEE platform through JavaScript programming^46^.

#### RSEI model construction

RSEI is an index used to assess terrestrial ecological quality. The existence of a significant amount of water body information can impact the humidity indices and potentially distort the results in the subsequent principal component analysis. As a result, it is essential to mask out the water body information within the study area prior to constructing the model. The Modified Normalized Water Index (MNDWI)^47^ in Eq. (8) is utilized to find and mask the water body information in the study region. Also, because the scale difference between the four ecological indicator components must be removed before conducting principal component analysis, this study normalized and standardized several indicators. The masked water and standardized indicators were subjected to Principal Component Analysis (PCA). If the contribution rate of the first Principal Component (PC1) is much greater than that of the other principal components, it means that PC1 contains most of the information, and then PC1 is selected as the initial $RSEI_{0}$^48^. $RSEI_{0}$ can be expressed in Eq. (9). If the eigenvectors of the NDVI and TCW indices have negative signs, reduction must be performed by subtracting PC1 from 1^27^.

8 $$\begin{array}{*{20}l} {MNDWI = \frac{{\left( {\rho_{Green} - \rho_{swir1} } \right)}}{{\left( {\rho_{Green} + \rho_{swir1} } \right)}}} \\ \end{array}$$

9 $$\begin{array}{*{20}l} {RSEI_{0} = \left\{ {\begin{array}{*{20}l} {PC1|\left[ {NDVI,\;TCW,\;NDBSI,\;LST} \right],} & {\quad NDVI > 0\; and \;TCW > 0} \\ {1 - PC1|\left[ {NDVI,\;TCW,\;NDBSI,\;LST} \right], } & {\quad NDVI < 0 \;and \;TCW < 0} \\ \end{array} } \right.} \\ \end{array}$$

where $\rho_{Green}$ and $\rho_{swir1}$ represent the B2 and B7 bands of the TM and ETM+ sensors, as well as the B3 and B7 bands of the OLI sensor. In Eq. (9), $RSEI_{0}$ represents initial RSEI, and *NDVI*, *TCW*, *NDBSI*, and *LST* represent eigenvectors of PC1.

In order to ensure the comparability and measurability of RSEI, Eq. (11) was adopted to normalize $RSEI_{0}$ within the range of 0 to 1 in this case. The closer the RSEI value is to 1, the better the ecological quality is. According to previous research, the RSEI results were classified into five levels: poor (0–0.2), fair (0.2–0.4), moderate (0.4–0.6), good (0.6–0.8), and excellent (0.8–1)^16^ (Xu et al. 2019)^48^.

10 $$\begin{array}{*{20}l} {RSEI_{normalized} = \frac{{\left( {RSEI_{0} - RSEI_{0min} } \right)}}{{\left( {RSEI_{0max} - RSEI_{0min} } \right)}}} \\ \end{array}$$

where $RSEI_{normalized}$ represents normalized RSEI, $RSEI_{0}$ represents initial RSEI, $RSEI_{0min}$ represents the minimum value of initial RSEI, and $RSEI_{0max}$ represents the maximum value of initial RSEI.

#### Accuracy evaluation

Ecological indices evaluation: To assess the accuracy of the HANTS algorithm in reconstructing ecological indicator time series, we adjusted the parameter frequencies, $f_{i}$, within the HANTS algorithm. We then compared the annual correlation coefficients (R), root mean square error (RMSE) and standard deviation (STD) among the reconstructed indicators and the original indicators under different parameter settings. The calculation formulas are presented in Eqs. (12)–(14). Based on these evaluation indices, a Taylor diagram was constructed to determine the optimal parameters and validate the accuracy of the reconstructed indicators^49^.

11 $$R = \frac{{\mathop \sum \nolimits_{i = 1}^{N} \left[ {y_{i}^{o} - \overline{{y_{i}^{o} }} } \right]\left[ {y_{i}^{D} - \overline{{y_{i}^{D} }} } \right]}}{{\sqrt {\mathop \sum \nolimits_{i = 1}^{N} \left[ {y_{i}^{o} - \overline{{y_{i}^{o} }} } \right]\left[ {y_{i}^{o} - \overline{{y_{i}^{o} }} } \right]} }}$$

12 $$STD = \sqrt {\frac{1}{N}\mathop \sum \limits_{i = 1}^{N} \left( {y_{i}^{D} - \overline{{y_{i}^{D} }} } \right)^{2} }$$

13 $$RMSE = \sqrt {\frac{1}{N}\mathop \sum \limits_{i = 1}^{N} \left( {y_{i}^{o} - \overline{{y_{i}^{D} }} } \right)^{2} }$$

where $y_{ }^{o}$ represents the original indicators, $y_{ }^{D}$ represents the reconstructed indicators, and *i* = 1, 2, 3… *N*, with *N* as the time series length.

RSEI evaluation: To ensure the comprehensiveness of information and the accuracy of measurable evaluation in the reconstructed RSEI model, 10,500 sample points were randomly selected from 2000 to 2020, including 500 points. The variables were projected into a three-dimensional space to examine spatial correlation and determine whether there is a spatial correlation and its degree between the independent variable RSEI and the four ecological indices (dependent variables). In addition, to compare the differences between the RSEI models constructed based on the reconstructed indicators and those constructed based on the original indicators, the disparities between the reconstructed RSEI models and the RSEI models based on original indicators for the years 2000, 2012, and 2020 were calculated.

#### Ecological quality trend analysis

The Sen slope estimator and Mann–Kendall test (Sen + MK) are trend analysis methods that combine both Theil-Sen and Mann–Kendall methods to consider both linear and non-linear changes in time series data. Known for its high computational efficiency, insensitivity to measurement errors and outlier data, it is particularly suitable for analyzing long-term series data trends.

Sen is a non-parametric statistical method used for analyzing trends, as demonstrated in Eq. (14). This method is particularly effective in identifying trends in long-time-series data, as it is robust against measurement errors and outliers^50^.

14 $$\beta = mean\frac{{x_{j} - x_{i} }}{j - i},\quad \forall j > i$$

where *β* represents the value of Sen, $x_{j}$ and $x_{i}$ represent time series data points, where a value of *β* exceeding 0 suggests an increasing trend in the time series, while *β* below 0 signifies a declining trend.

The M–K test is a non-parametric statistical method that has the advantage of not requiring assumptions about data distribution, displaying strong robustness, and being unaffected by data scaling. It is particularly well-suited for assessing trends in time series data^51,52^. The calculations are provided in Eqs. (16)–(19):

15 $$Z_{MK} = \left\{ {\begin{array}{*{20}l} {\frac{S - 1}{{VAR(S)}}} & {\quad if\; S > \varepsilon } \\ 0 & {\quad if \;S \le \varepsilon } \\ {\frac{S - 1}{{VAR(S)}}} & {\quad if \;S < \varepsilon } \\ \end{array} } \right.$$

16 $$S = \mathop \sum \limits_{j = 1}^{n - 1} \mathop \sum \limits_{i = j + 1}^{n} (x_{i} - x_{j} )$$

17 $$\begin{array}{*{20}l} {sgn(x) = \left\{ {\begin{array}{*{20}l} 1 & {\quad if} & {\left| {x_{i} - x_{j} } \right| > \varepsilon } \\ 0 & {\quad if} & {\left| {x_{i} - x_{j} } \right| \le \varepsilon } \\ { - 1} & {\quad if} & {\left| {x_{i} - x_{j} } \right| < \varepsilon } \\ \end{array} } \right.} \\ \end{array}$$

18 $$VAR(S) = \frac{1}{18}\left( {n\left( {n - 1} \right)\left( {2n + 5} \right) - \mathop \sum \limits_{k = 1}^{p} q_{k} \left( {q_{k} - 1} \right)\left( {2q_{k} + 5} \right)} \right)$$

where* S* represents the statistic, which is obtained by summing $sgn(x)$, $VAR(S)$ represents the variance, $n$ denotes the quantity of data entries, $Z_{MK}$ is the statistic for the Mann–Kendall test, and $q_{k}$ represents the count of identical data groups. The Sen slope estimation and the Mann–Kendall test in this study were conducted using ArcGIS Pro software^53^.

In order to determine the turning year of ecological quality changes, first evaluate the overall trend of ecological quality from 2000 to 2020, then calculate the sum of the cost functions (Eq. 19) at the intersection and use at least 3 consecutive data points to establish a piecewise linear regression equation. The smaller the sum of the cost functions, the smaller the difference between the predicted value and the true value.

19 $$J = \frac{1}{2m}\mathop \sum \limits_{i = 1}^{m} \left( {h_{\theta } \left( {x^{i} } \right) - y^{i} } \right)^{2}$$

$J$ represents the sum of cost functions, $m$ is the total number of samples, $x$ represents the input variable, $y$ represents the output corresponding to the input $x$ in the data set, and $h_{{\uptheta }}$ represents the input function.

#### Spatial correlation analysis

Spatial autocorrelation analysis is a statistical technique employed to evaluate the distribution properties and correlation of spatial data^54^. Examining the spatial autocorrelation of ecological quality helps reveal its distribution trends and interrelationships throughout the entire study area^55^. Moran’s I is a statistical method used for spatial data analysis, primarily for measuring geospatial correlations. It can be divided into global Moran index and local Moran index. Both indices are commonly used tools in spatial data analysis to understand the relationship between geographical phenomena and spatial data. This study utilizes global and local Moran indices to scrutinize the spatial correlation of RSEI ratings. Global spatial autocorrelation considers the spatial interrelation between different locations across the entire study area. It is useful for analyzing and depicting the overall spatial distribution pattern of the region, offering statistical inference on the overarching spatial pattern and facilitating comprehensively understand the spatial structure and correlations throughout the region. The *GlobalMoran*’*s* *I* in Eq. (19) quantifies the level of correlation between neighboring spatial unit values. A higher Moran's I value indicates stronger spatial autocorrelation. Strong spatial correlation in the overall spatial pattern suggests a certain regularity in the spatial distribution of RSEI. The local Moran index can further analyze the spatial correlation of RSEI and reveal localized patterns within the overall spatial pattern. This can help identify areas within the region with specific characteristics or unique circumstances^56^. The *LocalMoran*’*s* *I* in Eq. (20) represents the local Moran index, which describes the relationship between ecological quality among different grid units in the study area^57^. The Local indicators of spatial association (LISA) cluster diagram, generated using the local Moran index, categorizes local spatial clustering into five types: high–high (HH), low–high (LH), low–low (LL), high–low (HL), and non-significant.

20 $$GlobalMoran{\text{'}}s \;I = \frac{{m \times \mathop \sum \nolimits_{i = 1}^{m} \mathop \sum \nolimits_{j = 1}^{m} W_{ij} \left( {D_{i} - \overline{D}} \right)\left( {D_{j} - \overline{D}} \right)}}{{\mathop \sum \nolimits_{i = 1}^{m} \mathop \sum \nolimits_{j = 1}^{m} W_{ij} \left( {D_{i} - \overline{D}} \right)^{2} }}$$

21 $$\begin{array}{*{20}l} {LocalMoran{\text{'}}s\;I = \frac{{\left( {D_{i} - \overline{D}} \right) \times \mathop \sum \nolimits_{j = 1}^{m} W_{ij} \left( {D_{j} - \overline{D}} \right)}}{{\mathop \sum \nolimits_{i = 1}^{m} \left( {D_{i} - D} \right)^{2} }}} \\ \end{array}$$

In Eqs. (19) and (20), *m* denotes the total of elements; $D_{i}$ signifies the ecological quality value located on *I*; $\overline{D}$ stands for the mean ecological quality value across all elements in the study region, and $W_{ij}$ for the spatial weight.

#### Analysis of driving factors of ecological quality

Geographic detectors are a set of statistical methodologies used to identify geographical variations and reveal the mechanisms that instigate the differences^58^. They include four detectors: factor detectors, interaction detectors, danger area detectors and ecological detectors. This study selected eight factors related to ecological quality as independent variables: average annual temperature (X1), average annual precipitation (X2), slope (X3), slope aspect (X4), altitude (X5), forest area ratio (X6), construction land area ratio (X7), and cultivated land area ratio (X8). The factor detector and interaction detector are used to detect the impact of natural and economic factors on ecological quality.

Factor detector: Detect factor X's contribution to explaining spatial variation in variable Y. Use *q*-values to measure:

22 $$\begin{array}{*{20}l} {q = 1 - \frac{{\mathop \sum \nolimits_{h = 1}^{L} N_{h} \sigma_{h}^{2} }}{{N\sigma^{2} }} = 1 - \frac{SSW}{{SST}}} \\ \end{array}$$

where *h* = *1 *… *n*, *L* is the strata of variable *Y* or factor variance. *SSW* and *SST* represent variances within a layer and the total variance of the entire area, respectively. The value of *q* is from 0 to 1.

Interaction test: Determine if factors X1 and X2 enhance or diminish the ability to explain the dependent variable Y when they interact. The method of evaluation is to first compute the q-values of components X1 and X2 for Y, then compute their interaction for comparison. The association between two factors can be categorized as shown in Table 3.

**Table 3:** Geographic detector interaction factor level judgment.

Judgments based	Interaction type
*q(X1 ∩ X2)* < *Min(q(X1), q(X2))*	Nonlinear weakening
*Min(q(X1), q(X2))* < *q(X1 ∩ X2)* < *Max(q(X1), q(X2))*	Single factor nonlinear weakening
*q(X1 ∩ X2)* > *Max(q(X1), q(X2))*	Two-factor enhancement
*q(X1 ∩ X2)* = *q(X1)* + *q(X2)*	Independent
*q(X1 ∩ X2)* > *q(X1)* + *q(X2)*	Nonlinear enhancement

*Min(q(X1),q(X2)): Take the minimum value among q(*X1*), q(X2),*Max(q(X1),q(X2)):* Take the minimum value among q(*X1*), q(*X2*) ), *q(X1)* + *q(X2)*: the sum of the two, *q(X1 ∩ X2)*: the interaction of the two.

## Results

### RSEI of the different vegetation seasons

**Figure 3:**
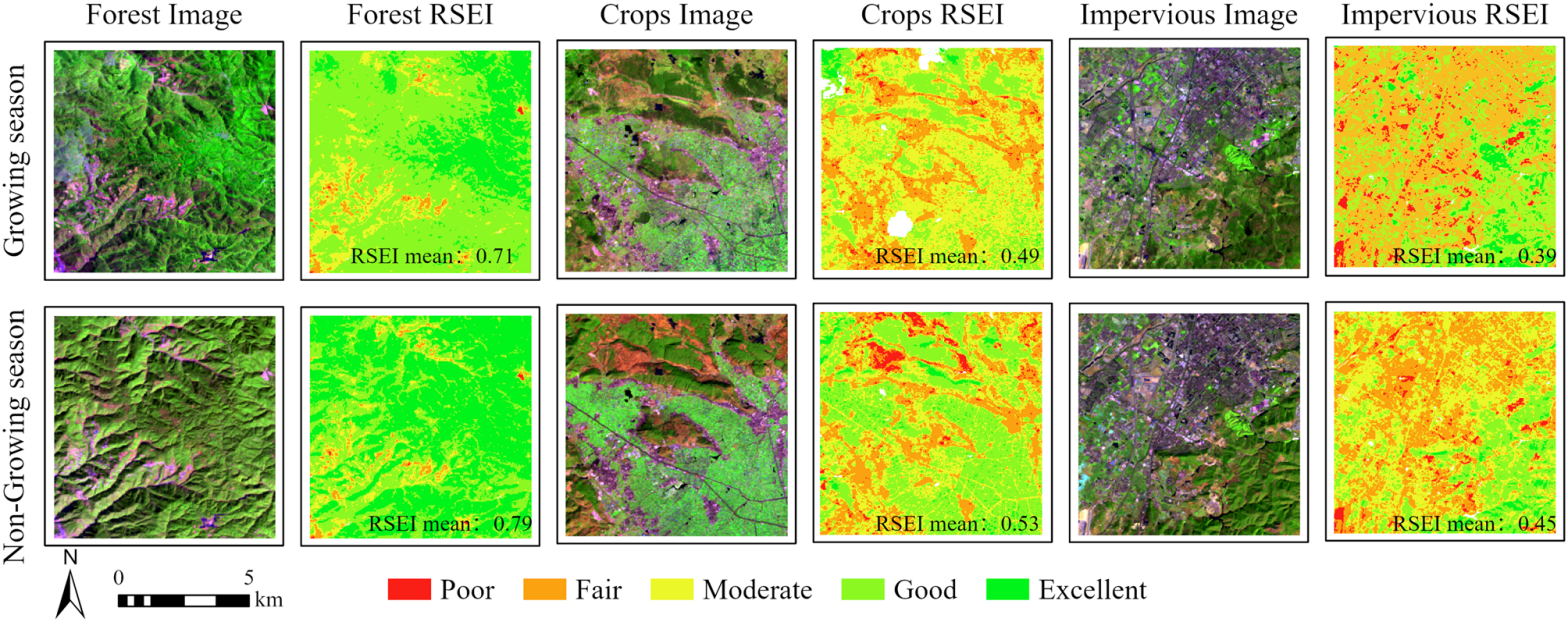
Comparison of RSEI of various species in the vegetation growing season and non-growing season. The map is designed using ArcGIS Pro V2.5 software provided by the ESRI website (https://www.esri.com/en-us/arcgis/products/arcgis-pro/overview). The images of forests, crops, and impervious during the growing and non-growing seasons are provided by the United States Geological Survey (USGS) (https://www.usgs.gov) Landsat 8 OLI imagery, available for free through Google Earth Engine (https://earthengine.google.com). The Remote Sensing Ecological Index (RSEI) is calculated using Google Earth Engine.

In order to analyze the differences in RSEI between plant growth period and the non-plant growth period, vegetation growth period data (May–October 2020) and data outside the vegetation growth period (November 2019–April 2020) were selected from Yuxi to establish RSEI for each period. The RSEI results of land cover, namely forest, cropland and impervious land were compared (Fig. 3). There are changes in the forest greenness information in satellite photos varies between growing and the non-growing periods. The greenness during the growth period is significantly higher than that during the non-growth phase; however, compared to the RSEI value of 0.71 in the growing season, the forest RSEI value in the non-growing seasons is higher, at 0.79. There is a large-volume crop information in the farmland images in both periods, but the greenness in the growing season is significantly higher than that in the non-growing season. However, the RSEI ratings for non-growing season farmland are concentrated at good (RSEI mean: 0.53), while the RSEI ratings for non-growing season are moderate (RSEI mean: 0.49). From the perspective of impermeable images, the urban information of the two periods remains consistent, but from the RSEI results, the score during the non-growth phase (RSEI mean: 0.45) is apparent higher than that during the growth phase (RSEI mean: 0.39).

### Ecological indices fitted by HANTS

**Figure 4:**
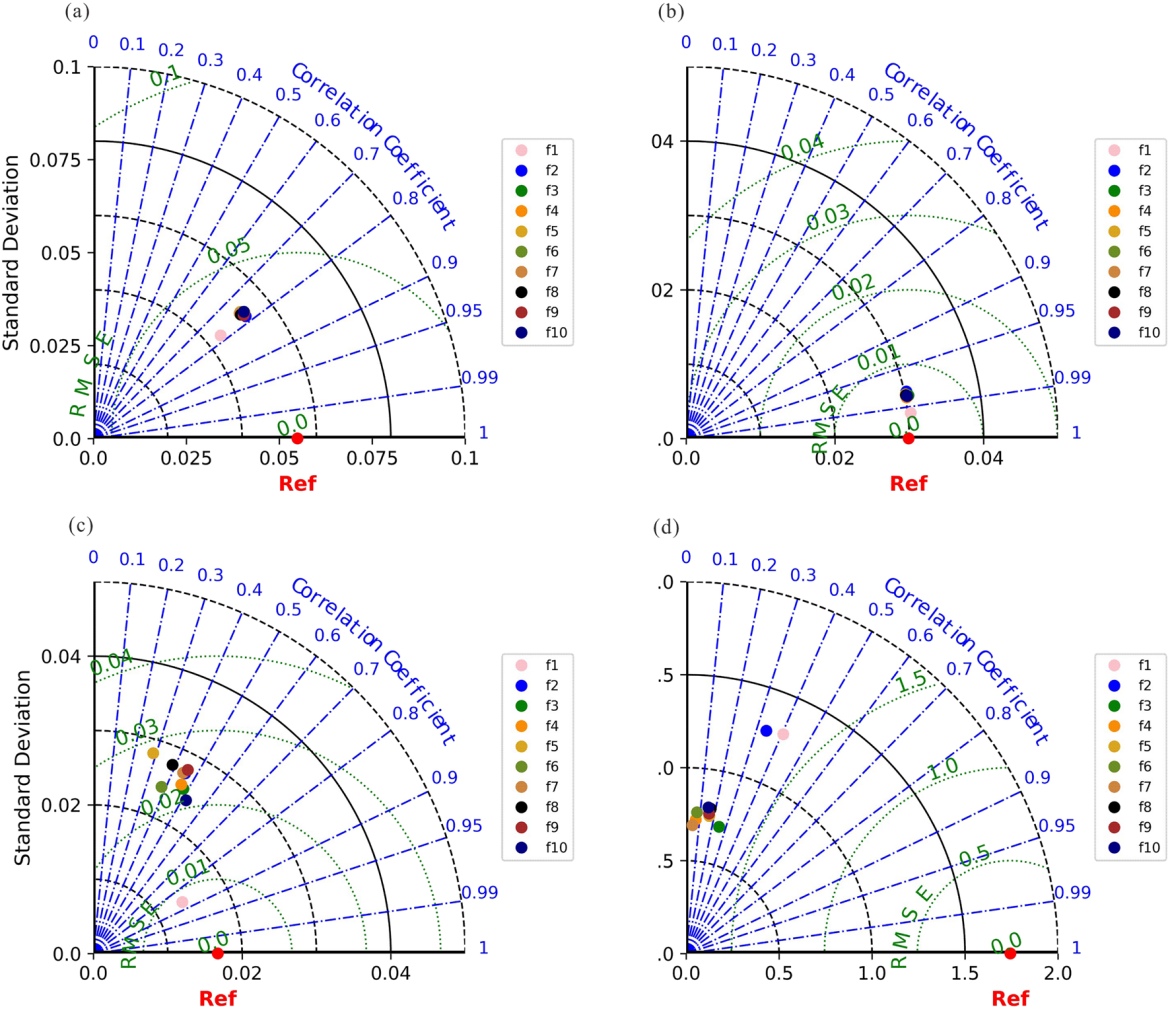
Reconstruction—Accuracy comparison of original ecological indices ((**a**–**d**) respectively correspond to the accuracy of the reconstructed *NDVI*, *TCW*, *NDBSI*, *LST* indices and each original indicator under different HANTS parameter (f1–f10) settings).

The HANTS frequency parameter $f_{i}$ is adjusted between 1 and 10, and ten models for each ecological indices are constructed to form a Taylor diagram (Fig. 4). The HANTS performs best when the parameter $f_{i}$ is set to 1, as the reconstructed values of the four ecological indicators are closest to the observed values (Ref). TCW and NDBSI rebuilt models performed best, with correlation coefficients R above 0.9 and RMSE and STD less than 0.01. Furthermore, the reconstructed NDVI demonstrated a high correlation, with R larger than 0.7 and both RMSE and STD less than 0.05. LST has an R of 0.4, and the RMSE and STD are around 1.5.

**Figure 5:**
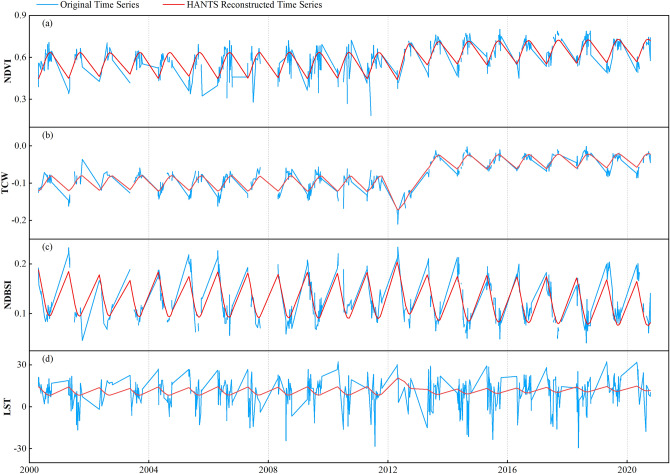
Original-reconstructed ecological indices time series comparison chart. (**a**–**d**) Represent NDVI, WET, NDBSI, and LST indices time series respectively.

To carry out a more comprehensive analysis on the usability of reconstructed indices, we proceeded to produce both reconstructed and original time series data for a total of 519 ecological indices images from May to October 2000 to 2020 (Fig. 5). The results of HANTS effectively eliminate noise, identified and resolved outliers in the dataset, and provide reasonable padding of missing data. Compared with the original sequence, the reconstructed indices sequence has a higher smoothness, which can more clearly represent the dynamic patterns exhibited by each of the four ecological indices. Among them, the reconstructed LST sequence eliminates the influence of extreme temperatures in the original sequence, making the sequence more average. This is also the reason why the reconstructed LST sequence is less accurate than the original LST sequence. The observed pattern of changes in the reconstructed ecological indices sequence are consistent with those in the original ecological index sequence, suggesting that the reconstructed sequence holds potential as a feasible foundation for the development of the RSEI model.

### RSEI model based on reconstruction indices

The annual were derived through principal component analysis of the median composite images spanning from May to October in the reconstructed index series (refer to Table 4). The contribution rates revealed that the lowest percentage was recorded in 2010 (66.32%), while the highest was observed in 2002 (87.24%). The average contribution rate stood at 78.52%, signifying that the primary principal component encapsulated the most significant ecological index information. Nevertheless, the feature vectors of each index indicated that the vector directions of NDVI and TCW, which were expected to have positive impacts on ecological quality, exhibited negative trends, contradicting the empirical evidence. Consequently, these vectors required adjustment by 1-PC1. Furthermore, the feature vector values highlighted that NDBSI held the most substantial proportion among the four indices, with LST comprising the least proportion. WET displayed relatively consistent values, whereas NDVI demonstrated fluctuations. This pattern suggested that aridity played a dominant role in influencing the ecological quality of Yuxi. The annual average values of RSEI demonstrated that the ecological condition of Yuxi was moderately favorable from 2000 to 2020, with a mean value of 0.5413. Analysis of the feature vector values indicated that RSEI values were lower in years characterized by diminished NDVI values. Conversely, RSEI values escalated in years marked by higher NDVI values, underscoring the pivotal role of greenness in determining ecological quality.

**Table 4:** ${RSEI}_{0}$ based on reconstructed ecological sequence.

Year	Eigenvectors				Contribution rate%	RSEI mean
	NDVI	TCW	NDBSI	LST		
2000	− 0.43	− 0.59	0.67	0.12	84.72	0.52
2001	− 0.44	− 0.52	0.61	0.38	70.03	0.54
2002	− 0.45	− 0.58	0.65	0.16	87.24	0.56
2003	− 0.42	− 0.57	0.65	0.26	86.22	0.55
2004	− 0.43	− 0.51	0.58	0.45	75.37	0.55
2005	− 0.37	− 0.54	0.61	0.43	70.11	0.54
2006	− 0.31	− 0.54	0.61	0.48	75.65	0.52
2007	− 0.34	− 0.53	0.60	0.47	74.99	0.54
2008	− 0.32	− 0.54	0.60	0.48	75.11	0.51
2009	− 0.29	− 0.55	0.61	0.47	75.35	0.52
2010	− 0.24	− 0.60	0.66	0.35	66.32	0.51
2011	− 0.23	− 0.57	0.63	0.45	73.46	0.50
2012	− 0.50	− 0.56	0.61	0.23	86.4	0.55
2013	− 0.46	− 0.52	0.57	0.42	79.47	0.52
2014	− 0.46	− 0.51	0.56	0.44	82.79	0.54
2015	− 0.47	− 0.53	0.57	0.40	78.91	0.55
2016	− 0.45	− 0.54	0.57	0.40	78.08	0.58
2017	− 0.46	− 0.52	0.56	0.43	82.9	0.55
2018	− 0.44	− 0.53	0.56	0.44	81.12	0.53
2019	− 0.45	− 0.53	0.56	0.43	81.2	0.54
2020	− 0.47	− 0.52	0.56	0.43	83.52	0.56
Mean	− 0.40	− 0.54	0.60	0.39	78.52	0.54

**Figure 6:**
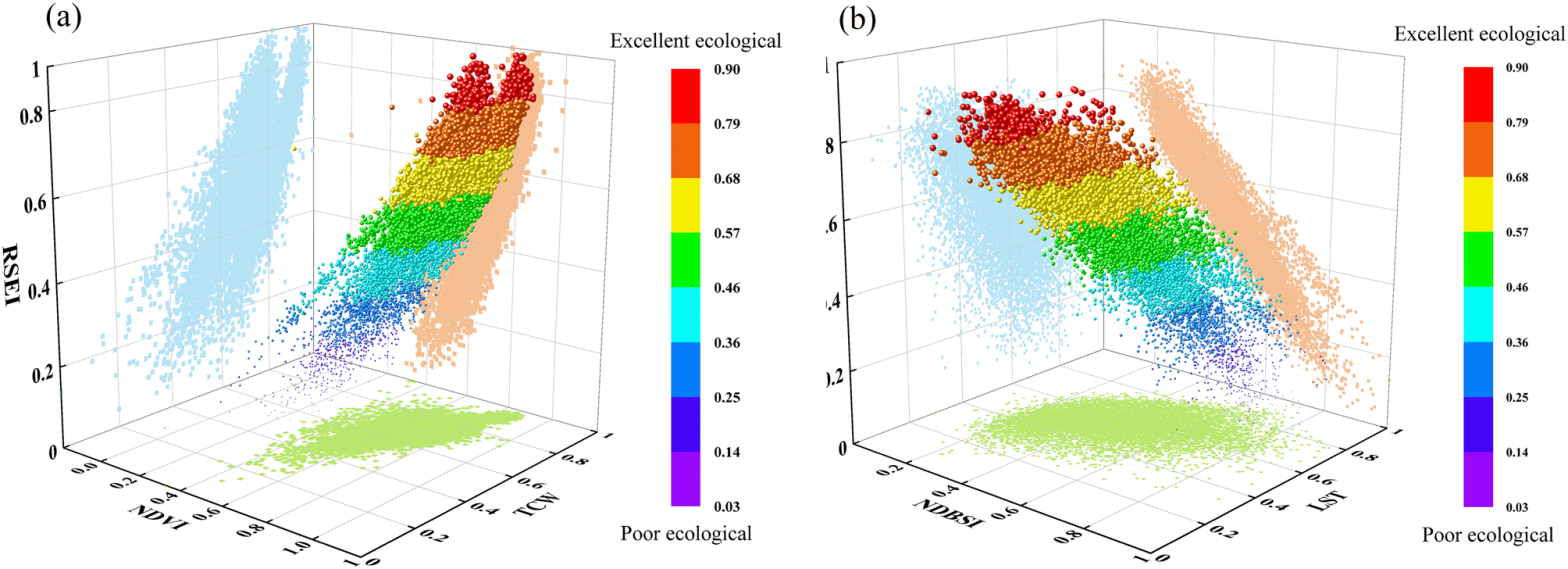
3D diagram of the relationship between each ecological indices and the RSEI Model. (**a**) Indicates the relationship between NDVI, TCW indices and RSEI. (**b**) Indicates the relationship between NDBSI, LST indices and RSEI.

The 10,500 sample points that were chosen were assigned the four ecological indices and the RSEI values for each year. These points were then projected onto a 3D space (Fig. 6) in order to analyze the link between each of the four ecological indices and RSEI. The ecological quality level is represented by the height of the scatter points in the picture, where good ecological quality is shown at the top and bad ecological quality is shown at the bottom. The objective laws are in line with the findings that greenness and wetness have a positive impact on ecological quality, while dryness and temperature have a negative effect. Figure 6a shows a positive correlation between NDVI and TCW and RSEI, while Fig. 6b shows a negative correlation between NDBSI and LST and RSEI. The scatter points also exhibit good aggregation, suggesting that the ecological indicator components can be taken into account by the reconstructed RSEI model, which can be utilized as a foundation for assessing ecological quality.

**Figure 7:**
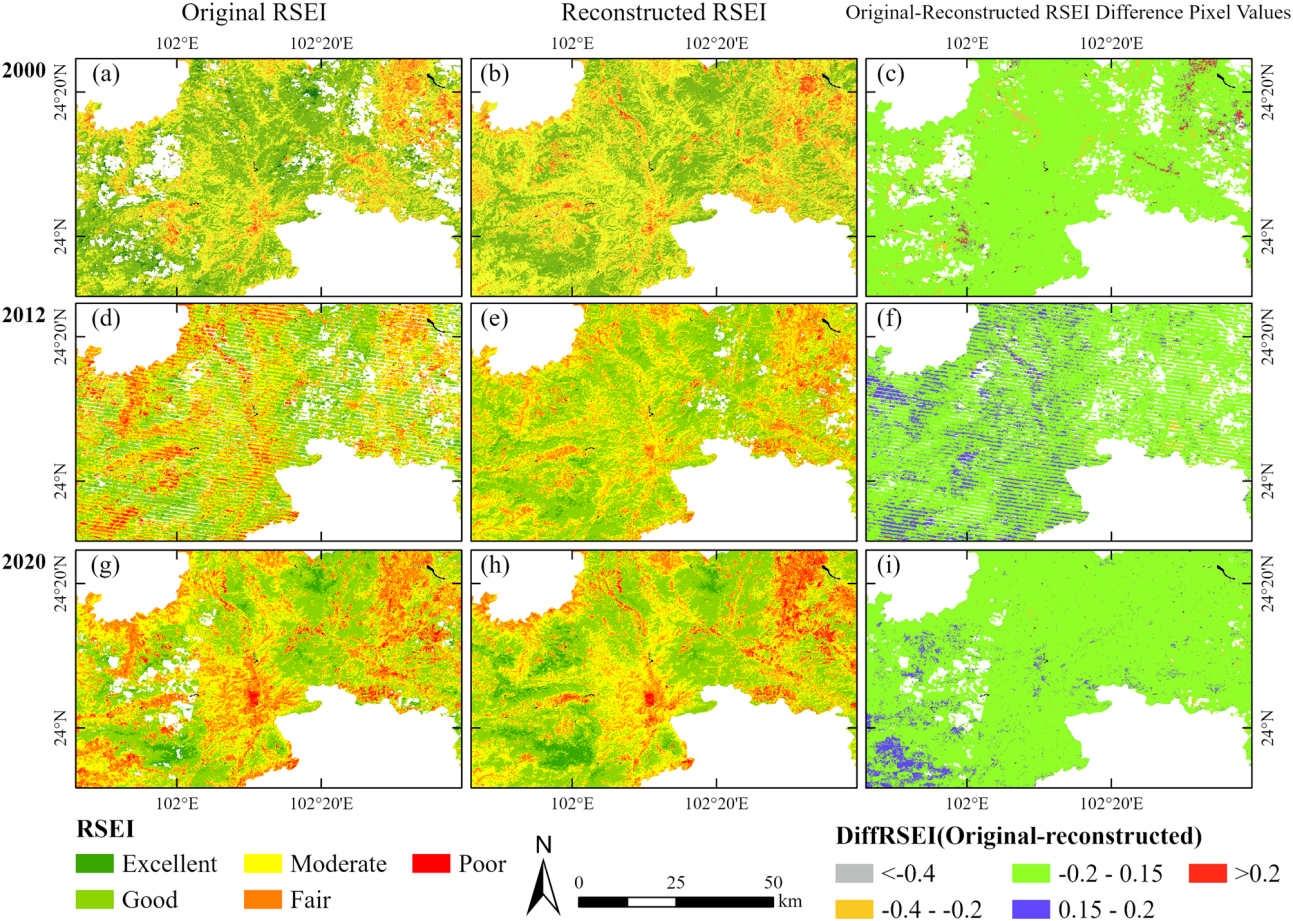
Calculate the absolute difference between the reconstructed RSEI model and the original RSEI model, where (**a**), (**d**), and (**g**) represent the original RSEI for the years 2000, 2012, and 2020, respectively, (**b**), (**e**), (**h**) represent the reconstructed RSEI index. The map is designed using ArcGIS Pro V2.5 software provided by the ESRI website (https://www.esri.com/en-us/arcgis/products/arcgis-pro/overview). Among them, the RSEI for the years 2000, 2012, and 2020 is calculated using the Landsat 5, 7, and 8 surface reflectance products provided by the USGS (https://www.usgs.gov). These products are freely available on Google Earth Engine (GEE) (https://earthengine.google.com), and the calculation process is also conducted within GEE.

Here is the corrected text: By comparing the difference between the RSEI based on the HANTS reconstruction indices and the original RSEI, this study validates the effectiveness of the RSEI by using reconstructed indices. The reconstructions from three time periods (2000, 2012, and 2020, representing TM, ETM+, and OLI sensors respectively) are compared with the original model. To improve the clarity of observation, the results of different models are magnified at the same scale in the central area of the image, with the study area center as the focal point of observation. Figure 7 shows the gap between the original RSEI and the reconstructed RSEI. The results reveal that the initial model was influenced by the noise and cloud removal technique, resulting in missing and muddled RSEI information. The RSEI created by the reconstructed indicator based on HANTS filtering, on the other hand, filled the missing data area and removed the noise impact in the ecological indicator, thus increasing the quality of the RSEI image and more accurately portraying the ecological quality. According to the absolute difference analysis, the difference between the reconstructed and original models is primarily dispersed between − 0.15 and 0.15. This suggests that the RSEI model, which is generated by using ecological indicators reconstructed by the HANTS algorithm, is capable of effectively representing the information contained in the original RSEI model when applied to Landsat data.

### The spatiotemporal pattern of ecological quality in Yuxi

#### The temporal variations in ecological quality in Yuxi

**Figure 8:**
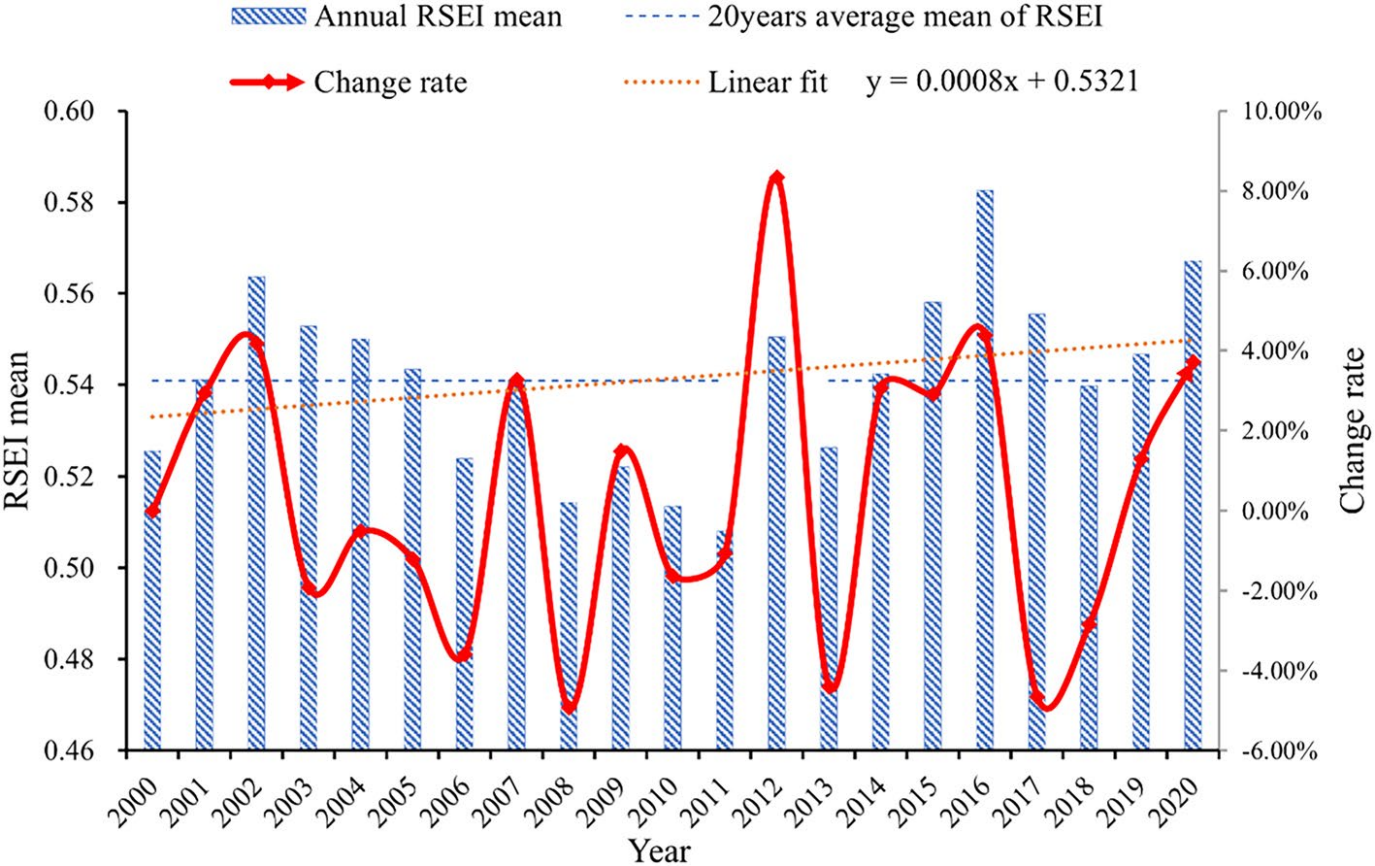
Changes in the average RSEI value of Yuxi each year from 2000 to 2020.

**Figure 9:**
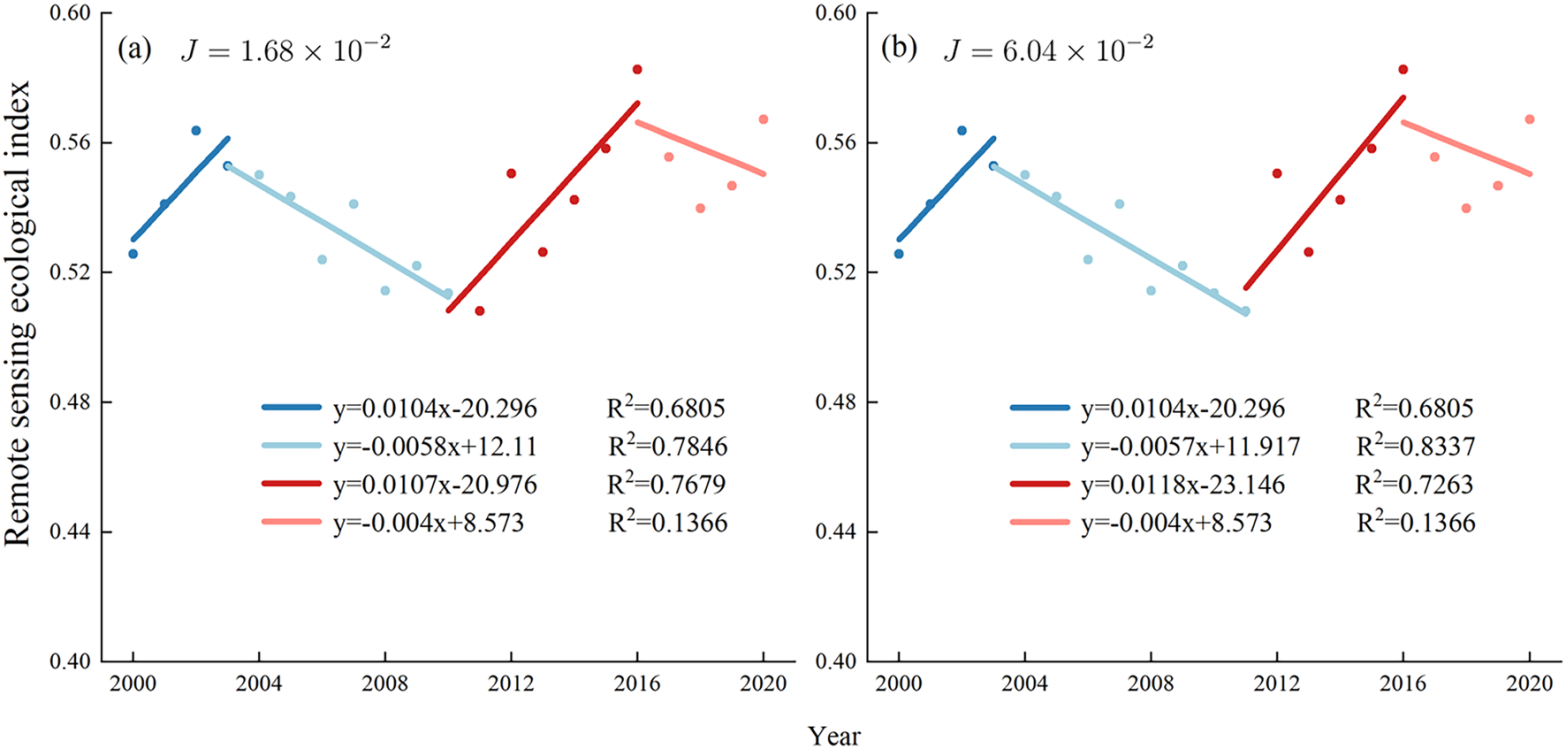
Trend of RSEI in Yuxi from 2000–2022. (**a**,**b**) represent two different piecewise regression strategies.

The RSEI change of Yuxi in the past 20 years is shown in Fig. 8. The RSEI fluctuates up and down, with a slight upward trend overall. In order to more objectively and comprehensively evaluate the spatiotemporal pattern of ecological quality in Yuxi over the last two decades (2000–2020), the turning point year of ecological quality change, i.e., the inflection point of RSEI, was first determined. The segmented linear regression model was used to construct two regression results (Fig. 9) by using the cost function, respectively. The R^2^ of the four segmented regression functions of the two regression models were similar, but the cost function J result of Fig. 9a was smaller than that of Fig. 9b. Therefore, we chose the result of Fig. 9a as the basis for the trend of ecological quality change in Yuxi. The turning point years of ecological quality change in Yuxi were 2003, 2010, and 2016, respectively. The ecological quality of Yuxi increased from 2000 to 2003, decreased from 2003 to 2010, and reached the lowest point; reached the highest point in 2016 and then began to decline.

#### The spatial variations in ecological quality in Yuxi

**Figure 10:**
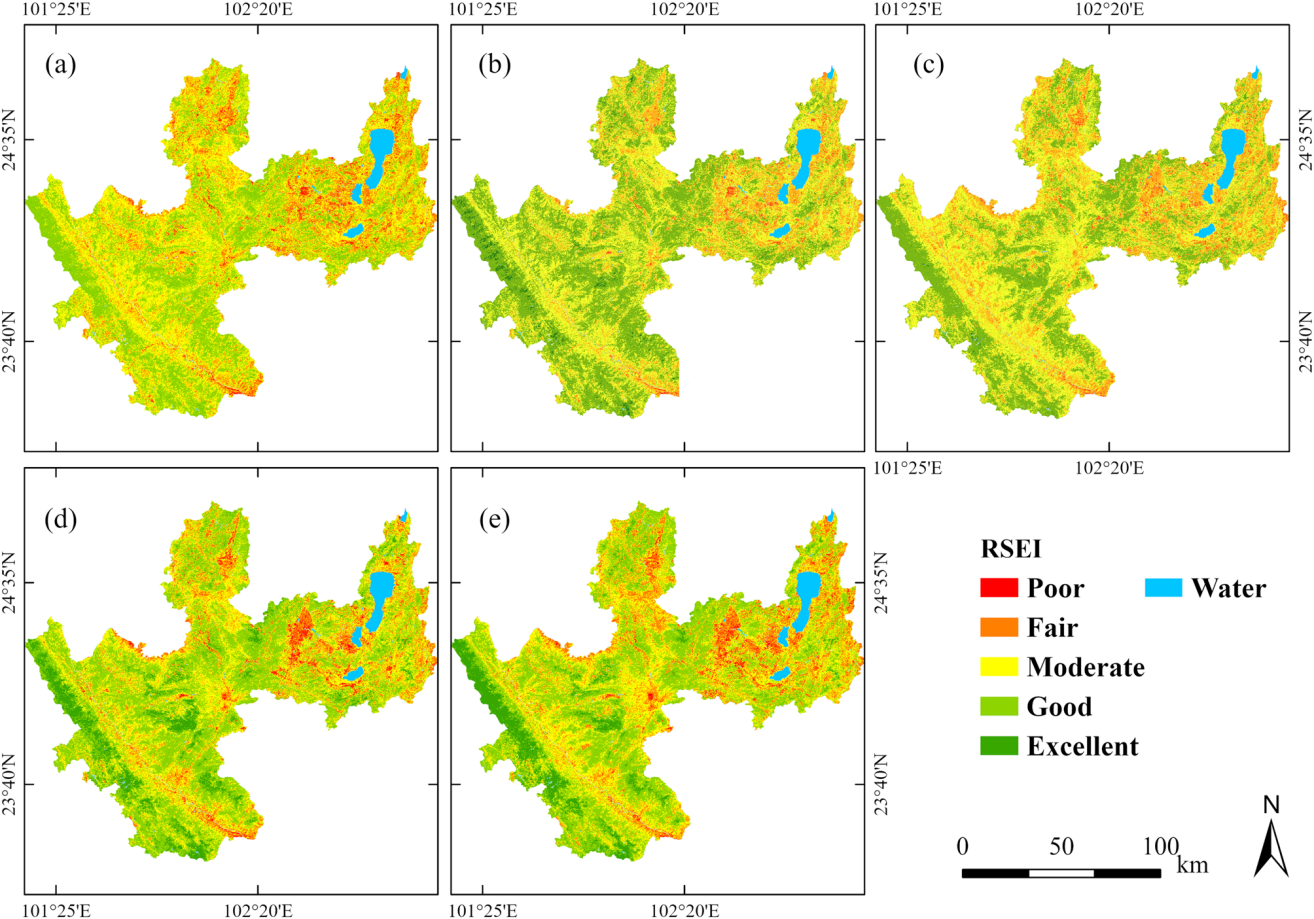
The content translation is the Spatial distribution of RSEI levels for different turning point years ((**a**–**e**) represent 2000, 2003, 2010, 2016, and 2020 respectively). The map is designed using ArcGIS Pro V2.5 software provided by the ESRI website (https://www.esri.com/en-us/arcgis/products/arcgis-pro/overview). RSEI is calculated using the Landsat series of satellites provided by the USGS (https://www.usgs.gov). Landsat 5 TM surface reflectance data was used in 2000, 2003, and 2010, while Landsat 8 OLI data was used in 2010 and 2020. Data calls and RSEI calculations are both implemented using Google Earth Engine (https://earthengine.google.com).

The RSEI series of Yuxi is classified into four phases based on the inflection points of RSEI changes, and the RSEI’s geographical distribution in Yuxi at the inflection years is depicted in Fig. 10. Geographically, in each transition year, the western portion of Yuxi has much greater overall ecological quality than the eastern region, and the mountainous regions in the west and central regions primarily have good and excellent grades. Lower ecological quality exists in the western valley, the middle and eastern plains, and the lands surrounding the lake, with rating levels ranging from moderate to poor.

**Figure 11:**
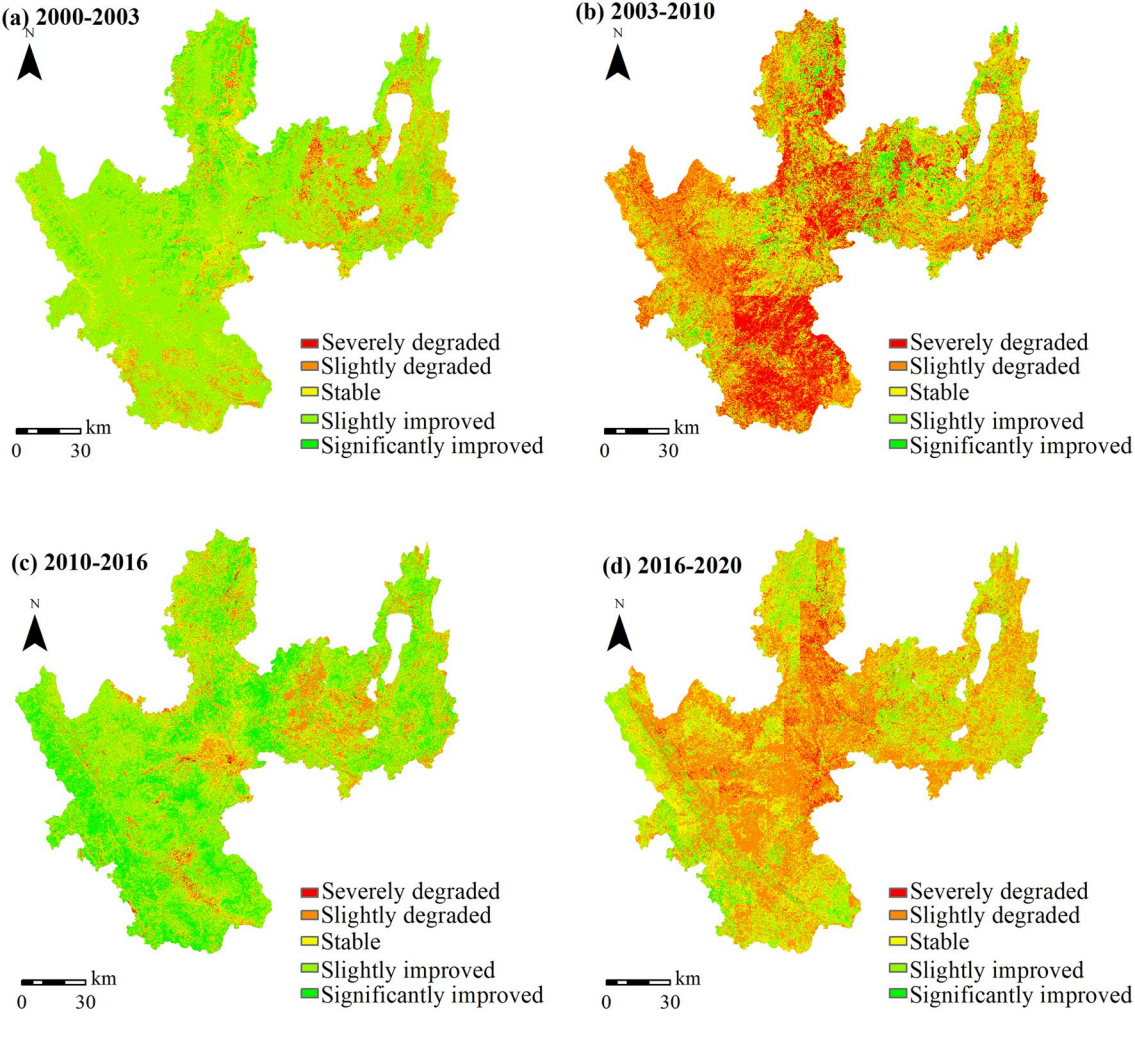
Change trends of RSEI in Yuxi at different stages. (**a**–**d**) represent the changing trends of RSEI in different stages. The map is designed using ArcGIS Pro V2.5 software provided by the ESRI website (https://www.esri.com/en-us/arcgis/products/arcgis-pro/overview). The satellite images used in the trend analysis are the Landsat series data provided by USGS (https://www.usgs.gov), among which Landsat 5 TM data were used from 2000 to 2010, Landsat 7 ETM+ data were used from 2012, and Landsat 8 OLI images were used from 2013 to 2020. The Sen + MK trend analysis was implemented using the Generate Trend Raster tool in ArcGIS Pro V2.5 software.

Sen trend analysis and the M–K test were performed on Yuxi's RSEI in four stages to better understand the ecological change characteristics (Fig. 11). The findings showed that the RSEI of Yuxi exhibited a clear increase trend from 2000 to 2003 (a decrease trend was only detected around lakes in the northeastern region). From 2003 to 2010, the RSEI in the lakes’ surrounding areas remained steady, with only a few locations continuing to fall. However, the RSEI fell dramatically in the central region, particularly in the plains west of the “Three Lakes” and in the districts surrounding the southern valley. The RSEI in Yuxi climbed dramatically between 2010 and 2016, but the RSEI in the central plains decreased significantly. The ecological condition of the entire region began to drop dramatically between 2016 and 2020, however some places around the “Three Lakes” showed major improvements. The four-stage trend chart reveals that RSEI variations in Yuxi have a cyclical rising and downward tendency, which is consistent with the piecewise linear regression model results (Fig. 8).

**Figure 12:**
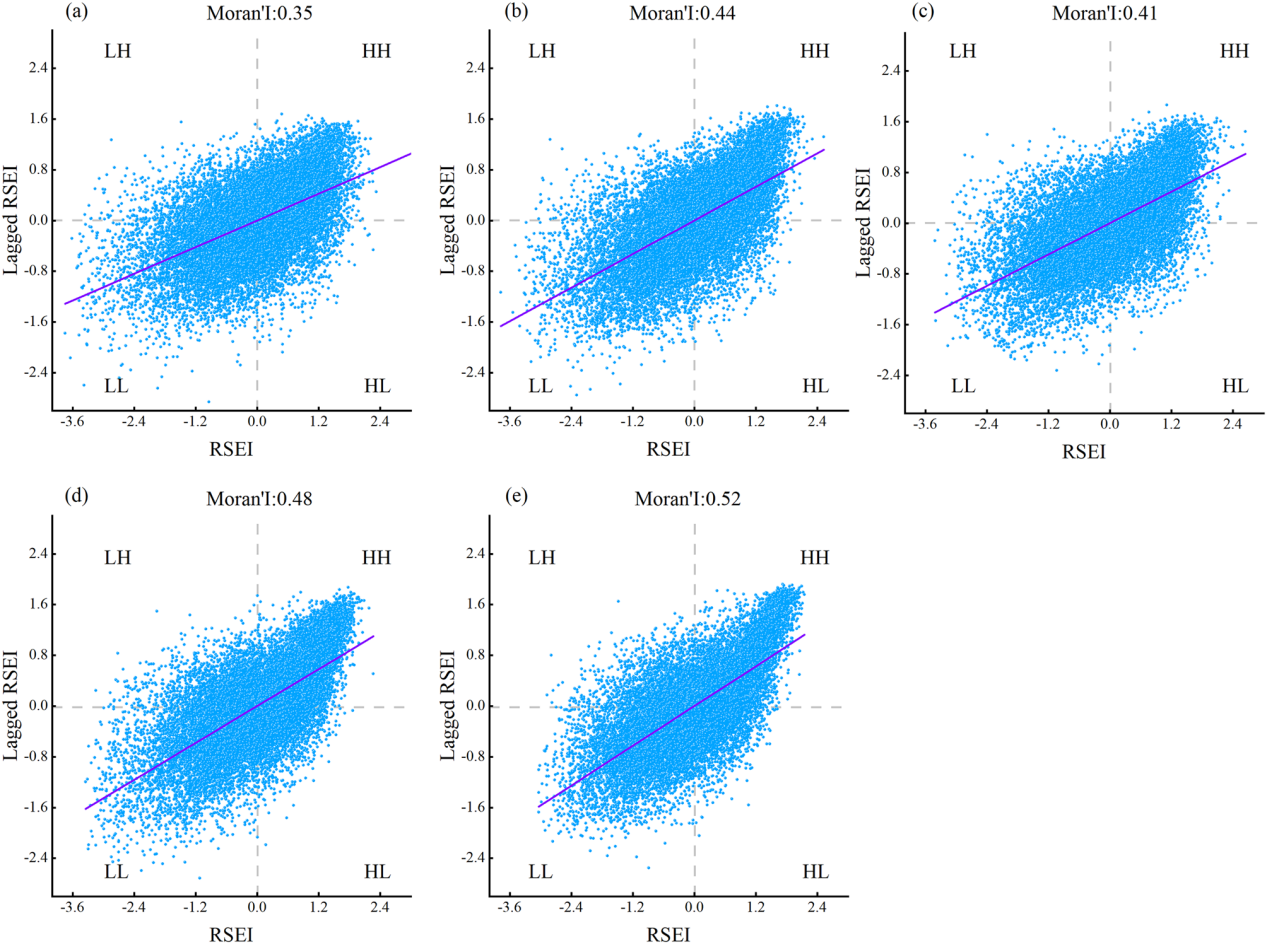
Moran I index of RSEI in Yuxi, (**a**–**e**) represent 2000, 2003, 2010, 2016, and 2020 respectively.

In order to confirm the spatial correlation of RSEI in Yuxi, spatial autocorrelation analysis was conducted on RSEI in the transition years using 1 km × 1 km fishnet point sampling. The vast majority of points are spread in the first and third quadrants, as seen in Fig. 12, and the mean value of *Moran*’*s I* at five time points is 0.44, indicating that the RSEI in research area has a robust positive spatial connection, and these values exhibit a clustered pattern rather than a random one. This implies that the regions with higher ecological quality have similar high ecological quality as their neighboring regions, or the regions with lower ecological quality have similar low ecological quality as their neighboring regions, and there is a certain association among them.

**Figure 13:**
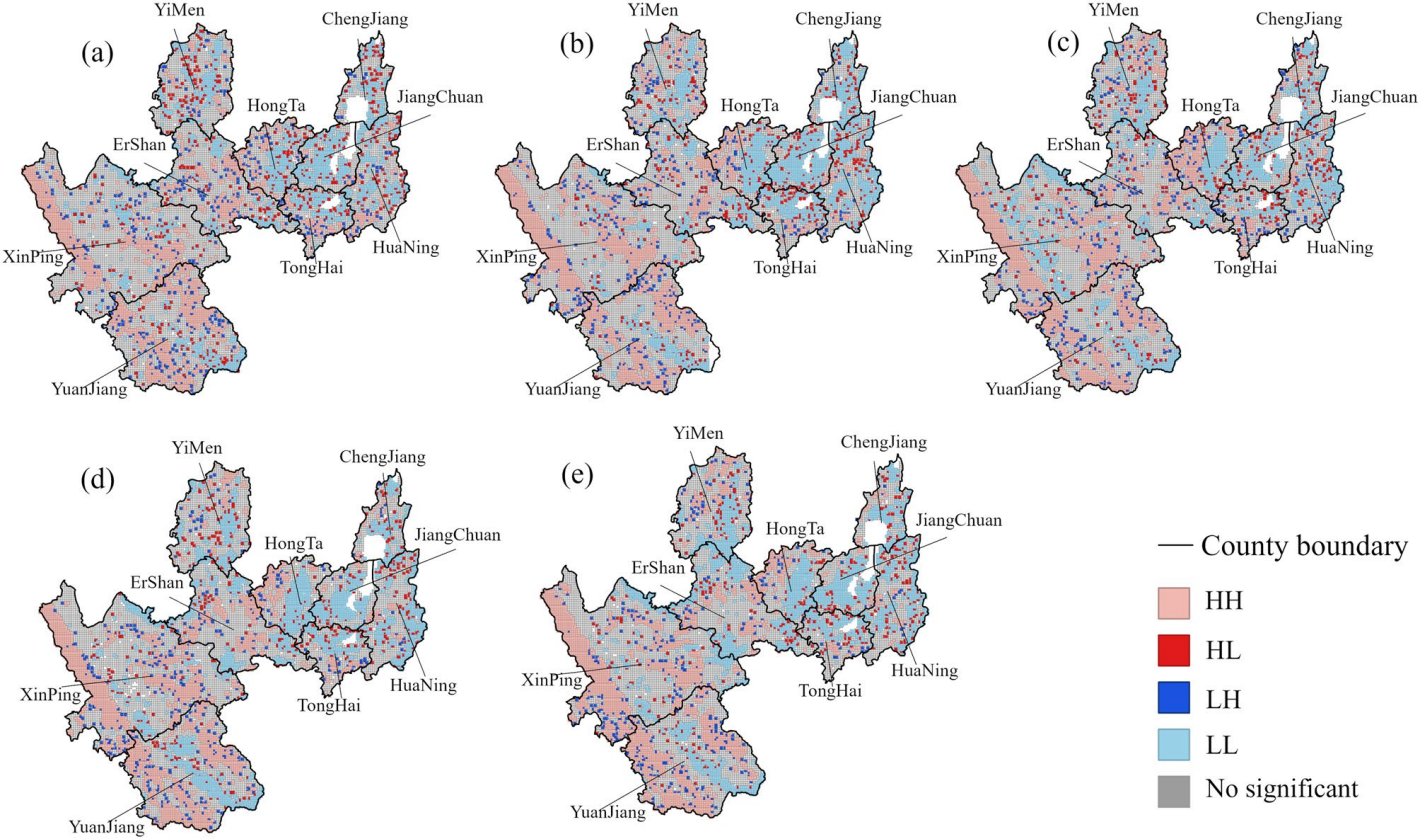
LISA analysis results, (**a**–**e**) represent 2000, 2003, 2010, 2016, 2020 respectively. The map is designed using ArcGIS Pro V2.5 software provided by the ESRI website (https://www.esri.com/en-us/arcgis/products/arcgis-pro/overview). The Landsat series of satellites provided by the USGS (https://www.usgs.gov). Landsat 5 TM surface reflectance data was used in 2000, 2003, and 2010, while Landsat 8 OLI data was used in 2010 and 2020. The local spatial autocorrelation map is implemented by the Cluster and Outlier Analysis tool in ArcGIS Pro V2.5 software.

The findings of a local spatial autocorrelation study on RSEI (Fig. 13) using LISA indicate that the majority of the unimportant areas at five time periods are located in the central and western valleys, as well as in hilly regions. The L–L cluster area is primarily dispersed throughout the plains and valleys, whereas the H–H cluster area is primarily found in the western and central mountains. This suggests that Yuxi’s ecological quality varies significantly across space, with high-quality ecological areas geographically isolated from low-quality ecological areas.

#### The spatiotemporal trends in ecological environment quality in Yuxi

**Figure 14:**
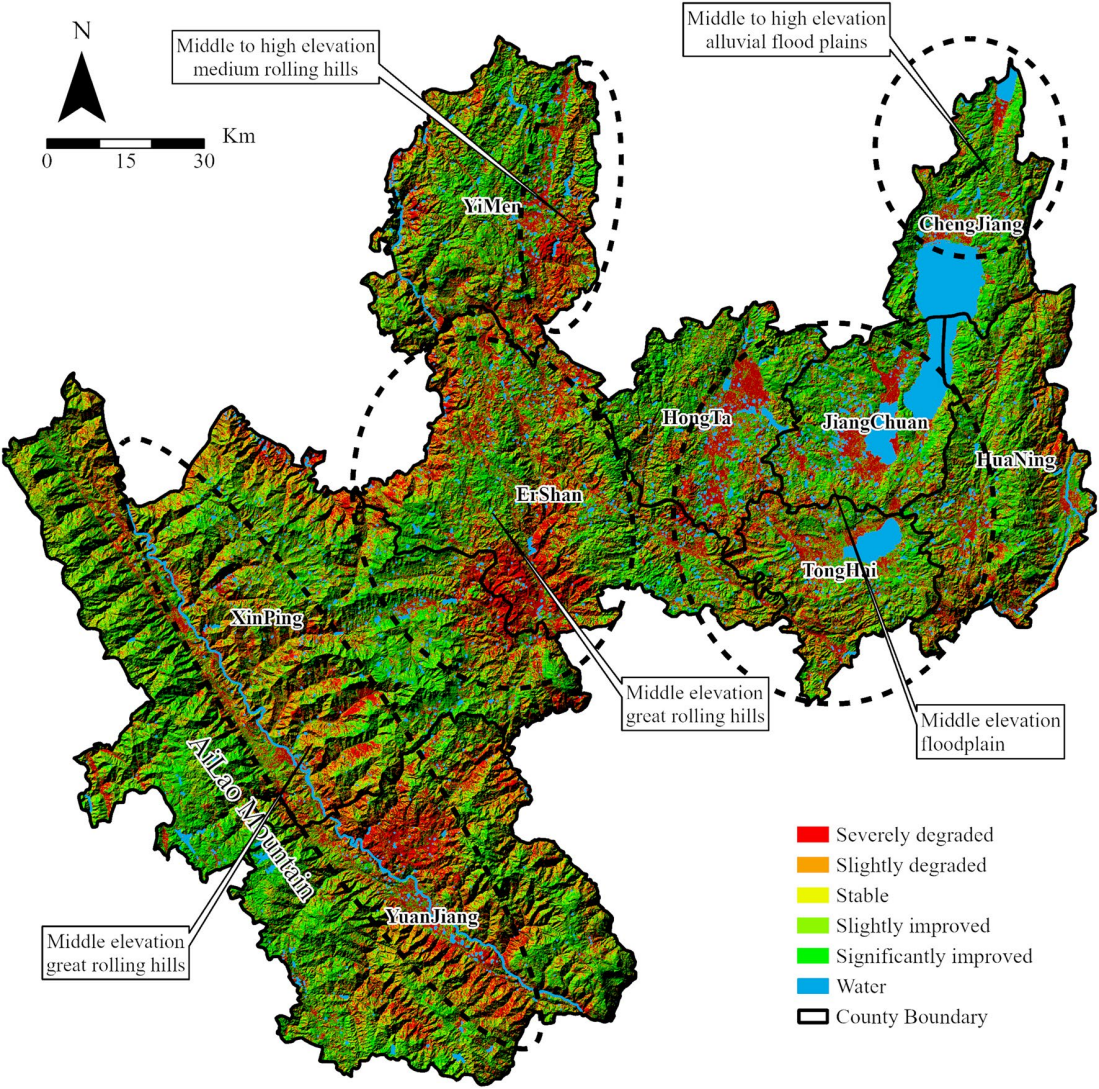
Sen + M–K analysis of RSEI in Yuxi from 2000 to 2020. The map is designed using ArcGIS Pro V2.5 software provided by the ESRI website (https://www.esri.com/en-us/arcgis/products/arcgis-pro/overview). The satellite images used in the trend analysis are the Landsat series data provided by UGCS, among which Landsat 5 TM data were used from 2000 to 2010, Landsat 7 ETM+ data were used from 2012, and Landsat 8 OLI images were used from 2013 to 2020. The Sen + MK trend analysis was implemented using the Generate Trend Raster tool in ArcGIS Pro V2.5 software.

Yuxi’s overall ecological quality has been gradually improving over the last 20 years, remaining at a moderate level. In particular, there has been an increasing trend and a declining trend in the change of ecological quality. The areas with ecological quality of ratings poor, fair, and moderate are primarily concentrated in the relatively flat plains and areas near water sources, according to the results in the spatial arrangement of ecological quality, whereas the areas with ecological quality ratings of good and excellent are concentrated in the steep mountains. Figure 14 displays the general RSEI trend changes. The areas with sharp drops in RSEI are concentrated in Xinping County, Yuanjiang County valley area, southeast of Yimen County, central Eshan County area, and near the “Three Lakes.” These areas are also relatively gentle positions or plains in large and medium undulating mountains, surrounded by mountains with large undulations. Furthermore, RSEI indicates a very strong increasing tendency in regions with substantial topographical undulations and a considerable distance from bodies of water. This suggests that both human activity and natural geographical factors have an impact on Yuxi's ecological quality. While the plains and valleys' ecological quality is more under pressure, the mountains and water sources contribute significantly to its improvement.

### Geographic detector results

#### Analysis of RSEI driver factor detection results

Table 5 shows that the driving variables of different eras have varying influences on Yuxi's RSEI. Forest proportion > impervious land proportion > slope ranked first among the driving factor q values from 2000 to 2003; similarly, forest proportion > impervious land proportion > altitude ranked first among the q values from 2010 to 2020; and forest proportion > impervious land proportion > precipitation ranked first among the driving factor q values from 2016 to 2020. In general, the primary factors influencing variations in RSEI are height, slope, precipitation, and the percentage of impervious land and forest.

**Table 5:** Geodetector single factor detection.

Driving factors		Temperature	Precipitation	Slope	Aspect	Altitude	Forest ratio	Cropland ratio	Impervious ratio
q	2000	0.03*	0.06*	0.07*	0.04*	0.03*	0.29*	0.01*	0.13*
	2003	0.03*	0.07*	0.09*	0.03*	0.05*	0.35*	0.01*	0.19*
	2010	0.05*	0.06*	0.06*	0.02*	0.09*	0.34*	0.01*	0.20*
	2016	0.06*	0.11*	0.08*	0.01*	0.11*	0.38*	0.03*	0.21*
	2020	0.08*	0.14*	0.08*	0.01*	0.13*	0.34*	0.02*	0.19*

*P < 0.05 for this item.

#### Analysis of RSEI driver factor interaction detection results

**Figure 15:**
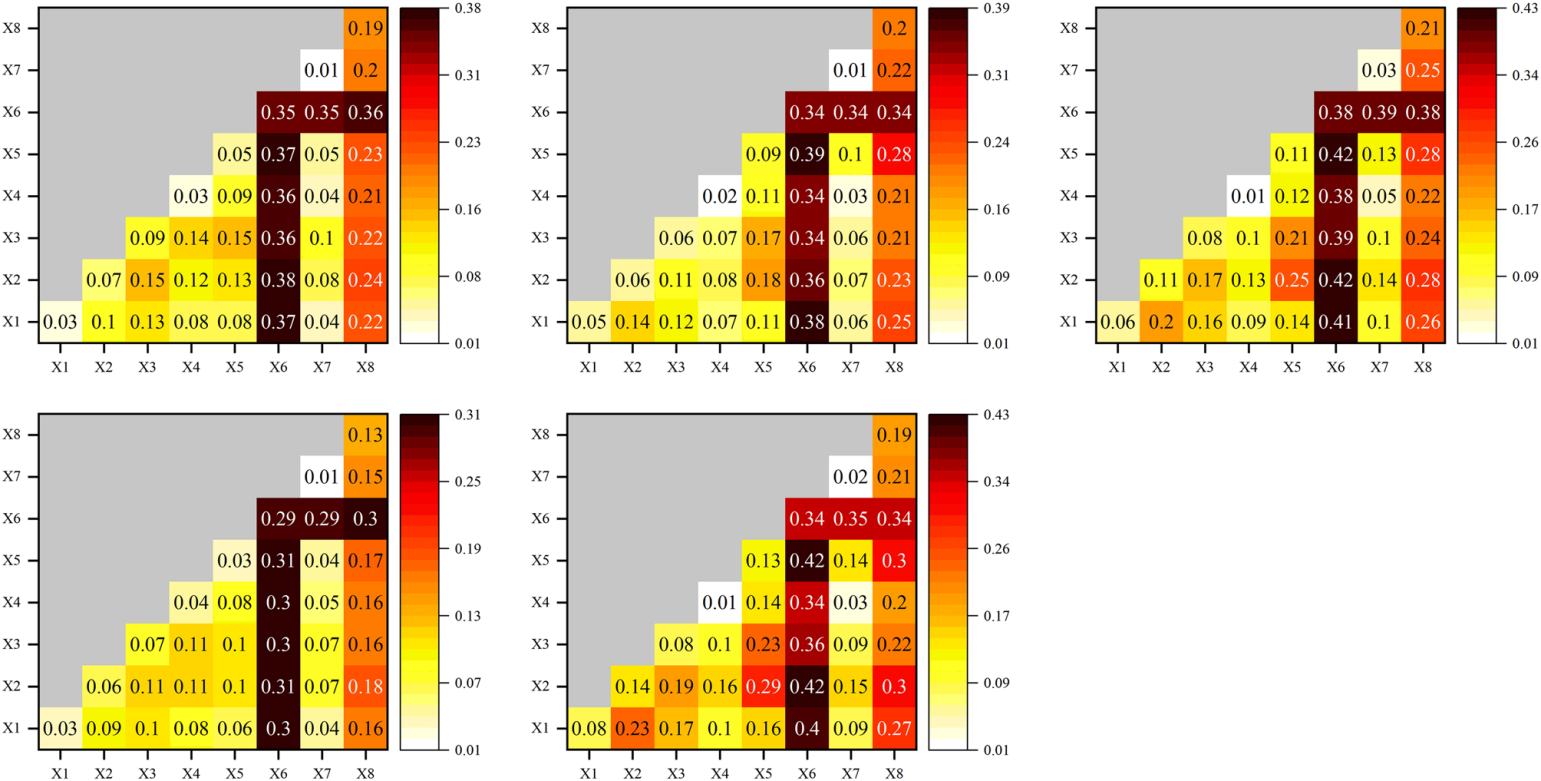
Geographic driver factor interaction detection results, (**a**–**e**) represent 2000, 2003, 2010, 2016, and 2020 respectively.

The results of the interaction are all nonlinear enhancements or double-factor enhancements, based on the findings from the interactive detection results of the RSEI driving factors in Yuxi from 2000 to 2020 (Fig. 15). This suggests that the interaction of two factors is primarily responsible for the alterations in the ecological quality of Yuxi. The interaction of eight factors has a higher explanatory power than the individual effects when compared to a single factor. From 2000 to 2020, the interaction of forest cover with other seven factors had the highest *q* value, which indicates that among the years when ecological quality showed a turning point, the interaction of forest cover with other factors had the greatest impact on the change of RSEI.

#### Changes in land use types in Yuxi

**Figure 16:**
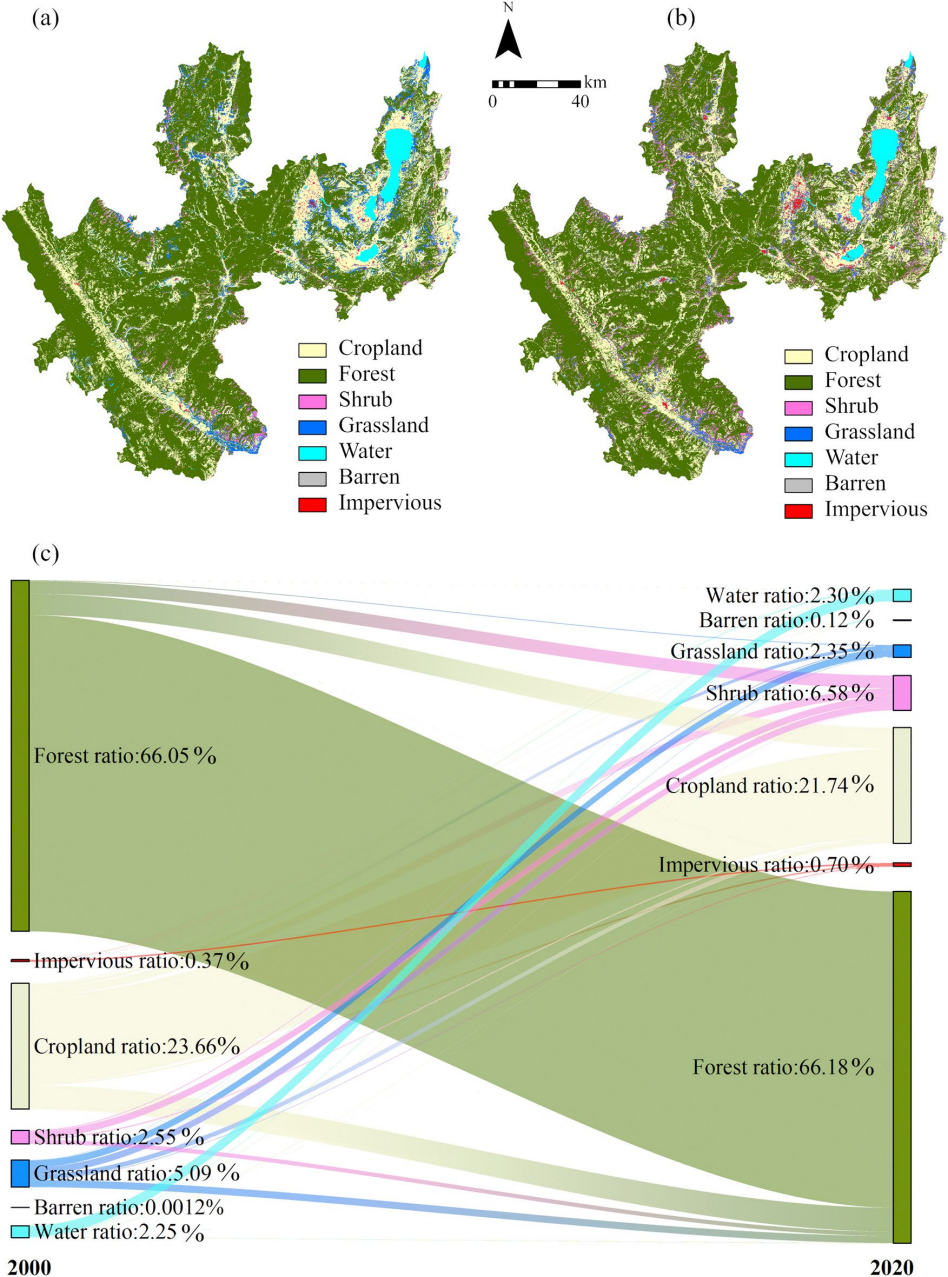
(**a**,**b**) are the land use maps of Yuxi in 2000 and 2020 respectively, and (**c**) is the land cover transition of Yuxi from 2000 to 2020. The map is designed using ArcGIS Pro V2.5 software provided by the ESRI website (https://www.esri.com/en-us/arcgis/products/arcgis-pro/overview). The land use classification results in 2000 and 2020 use the China land cover dataset (CLCD) (https://zenodo.org/).

The land use types in Yuxi in 2000 and 2020 were mapped, and a transition matrix was used to examine the changes in each land use type in order to explore the impacts of changes in land use types on ecological quality (Fig. 16). Yuxi’s land use types have changed dramatically during the previous 20 years. The total proportion of forest increased by 0.12%, but some forest also changed to shrub, grassland, and cropland; the proportion of impervious increased significantly, from 0.37 to 0.70%, indicating a trend of spreading from the central to the surrounding areas; the proportion of cropland decreased, with the majority of cropland changing to forest, shrub, and grassland, and a small amount of cropland changing to impervious; In summary, Yuxi has experienced tremendous urban expansion over the last 20 years, and there is a mutual conversion interaction between forest, cropland, grassland, and shrub. The proportion of forest has risen dramatically, whereas cropland and grassland have declined. These land use type changes are primarily influenced by economic development, population expansion, policy adjustments, natural disasters, and other variables, all of which affect Yuxi’s ecology differently. Among them, the expansion of impervious has the greatest negative impact on the ecological quality, leading to the depletion of land assets, biodiversity decline, aggravation of soil and water loss, the deterioration of environmental pollution and other problems; while the increase of forest and shrub has a greater positive impact on the ecological quality, it is conducive to improving vegetation coverage, enhancing the stability of ecosystems, reducing greenhouse gas emissions, improving climate conditions and other aspects.

## Discussion

### The vegetation growing season is an optimization time window for evaluating ecological quality by using RSEI

By comparing the RSEI constructed using two different vegetation seasonal windows, it reveals that the choice of time window significantly impacts the outcomes of RSEI analysis. The research highlights the vegetation growing season as the most appropriate time window for evaluating ecological quality through RSEI. In regions with distinct wet and dry seasons, the vegetation growing season is further subdivided into growing and non-growing seasons. Previous studies utilizing RSEI for ecological quality assessment have often included a substantial amount of non-growing season imagery to filter out high-quality image data, thereby neglecting the influence of seasonal windows on the construction^18,59,60^ of the RSEI model. Contrary to the commonly held belief that higher vegetation greenness indicates better ecological quality, this study uncovers that during the non-growing season, RSEI values for forests, farmland, and impervious surfaces are all elevated compared to the growing season (refer to Fig. 3). The reason for this result during the non-growing season, the weight of vegetation greenness information is lower, leading to RSEI overestimating regional ecological quality. Previous research has shown that vegetation condition is one of the key influencing factors in constructing RSEI and appropriate seasonal windows should be considered^17,48,61^. This study not only reiterates previous findings but also demonstrates that the vegetation growing season is the optimal time window for constructing RSEI. In conclusion, when using the RSEI model to evaluate regional ecological quality, the influence of vegetation seasons on model construction needs to be considered. The greenness information in the study area peaks during the vegetation growing season, more accurately reflecting the actual vegetation growth conditions in the study area. Therefore, when constructing RSEI models, incorporating vegetation growing season data into the selection of time windows enhances the accuracy and reliability of the assessment.

### HANTS is capable of optimizing the filling results for Landsat missing data

Meteorological conditions such as cloud cover, haze, atmospheric scattering, etc., can introduce noise into remote sensing imagery data, leading to discontinuities and incompleteness in specific areas and time periods^32^. The use of such data can severely affect the credibility of ecological quality assessment using Remote Sensing Ecological Index (RSEI). In previous studies, the construction of RSEI models typically relied on selecting the best quality image data for a given year or using interpolation methods to optimize the data, but this approach often ignored the temporal variations in building the RSEI^18,30,60^. This study employs the HANTS to fit ecological indices (NDVI, TCW, NDBSI, LST) for constructing RSEI. Compared to the original ecological index sequences, the ecological index sequences reconstructed based on HANTS fill in missing data, ensuring the wave form of the original sequence while providing smoother temporal curves. Due to the regularity of physical and biochemical characteristics during the vegetation growth season in the study area, the time series reconstructed by HANTS based on periodic behavior can consider the periodicity of the real-world production cycle^26,62^. Therefore, HANTS-reconstructed RSEI exhibits higher statistical correlation with the original RSEI at high-precision pixel level (absolute differences mainly concentrated between − 0.15 and 0.1), indicating that HANTS is more suitable for optimizing the time–frequency of comprehensive index RSEI. Previous studies have shown that when constructing RSEI using MODIS data, RSEI constructed based on HANTS performs better overall compared to SG and WS methods. This study further validates the applicability of HANTS in higher spatial resolution data. HANTS improves data quality while retaining temporal information in the data. In long-term time series analysis, this method can serve as an effective means of improving the quality of time series data.

### Evaluation and analysis of ecological quality distribution in Yuxi

This research conducted a spatial analysis of the RSEI and identified substantial spatial disparities in ecological quality within the Yuxi region. Regions with superior ecological quality are predominantly found within national ecological functional zones characterized by favorable natural conditions, robust vegetation coverage, and safeguarding under ecological redlines. These areas are primarily situated in mountainous and hilly terrains. In contrast, regions with inferior ecological quality are clustered in densely populated urban centers and agricultural lands near water sources, primarily located in plains and river valleys, demonstrating a distinct center-periphery distribution (with inferior ecological quality typically observed in the plain river valley areas surrounding towns, while higher ecological quality is observed in the mountainous areas encircling towns). This research finding is consistent with the results of studies by Geng, Lin, and others^16,17^. This phenomenon may be attributed to reduced human activities in mountainous and hilly regions, combined with environmental policy safeguards, leading to enhanced forest coverage and elevated greenness and humidity indices that positively influence ecological quality. In contrast, plain river valley areas experience denser human activities, with a large amount of construction and production activities leading to an increase in aridity index and consequently poorer ecological quality. Additionally, there is strong spatial correlation in ecological quality in Yuxi. High–high (H–H) cluster areas are mainly concentrated in the western and southern mountainous and hilly regions under environmental protection policies, while low–low (L–L) cluster areas expand outward from urban centers with the development of urbanization. In the future, ecological protection efforts in Yuxi should focus on areas near urban construction zones and strictly control the scale of urban development.

### Land type changes dominate the ecological quality of Yuxi

Land use type is the main factor affecting ecological quality, and changes in land use type are the dominant factor causing ecological quality (Table 4). In the past two decades, there has been a manifest trend of urban expansion in Yuxi. There is a mutual conversion relationship between woodland, farmland, grassland, and shrubs. The percentage of forestland has substantially risen, while the percentage of cropland and grassland has decreased (Fig. 15). Rapid urbanization has the greatest negative impact on ecological quality, while the increase in forests and shrubs has a more favorable effect on the ecological quality. The reason for this effect may be that the expansion of man-made surfaces leads to an increase in dryness information, while the increase in forest land increases greenness information. The changes in Yuxi’s ecological quality during the course of the last twenty years are closely related to the urbanization process and ecological protection policies. According to the Yunnan statistical Yearbook from 2000 to 2020, in 2000, the urbanization level of Yuxi was relatively low, with an urbanization rate of only 38.30%. Economic development was slow, but the Chinese government began to prioritize ecological civilization construction and green development, implementing a series of ecological projects. Therefore, during this period, the overall ecological quality of Yuxi significantly improved. However, since 2000, the urbanization development of Yuxi has been rapid. The proportion of impermeable surfaces increased from 0.37 to 0.70% (Fig. 16), and the urbanization rate reached 53.82% in 2020. The rapid urbanization has led to a rapid decline in the ecological quality of Yuxi. This is mainly because urban development has occupied a considerable portion of farmland and forests, resulting in land degradation and loss of biodiversity, while also increasing pollutant emissions and resource consumption. It is worth noting that since 2010, the effects of policies and ecological projects implemented by the Chinese government, such as returning farmland to forests, basin governance, and the construction of national-level nature reserves, have begun to show. The ecological quality of most mountains and lakes in Yuxi has improved significantly. However, due to rapid economic development, the ecological quality of major towns and some valleys is still declining. In the future, it is necessary to further strengthen ecological strategic planning and efficient management to achieve coordinated unity between promoting economic and social progress and protecting the ecological environment.

### Uncertainty analysis

There are still some potential areas for improvement. Although the HANTS has proven to be effective in creating composite indices and is widely used for time series reconstruction^63^, our work did not conduct a comparative analysis of other time series filtering techniques. Therefore, we are unable to quantify the optimal filtering approach. Comparative analysis of filtering algorithms has the potential to enhance the reliability of time series outcomes. Moreover, the choice of the surface temperature inversion algorithm could impact the generation of the Land Surface Temperature (LST) series. It is recommended for future research to explore different inversion techniques to evaluate and compare their performance differences in estimating LST. The data used in this study to create the time series was collected from Landsat 5/7/8 sensors. However, it is important to note that there are spectral differences among these sensors that cannot be completely eliminated. To improve data consistency, future research may consider prioritizing data from a single sensor to reduce uncertainties arising from spectrum disparities. This study presents significant findings on assessing the ecological quality of Yuxi. However, future research should concentrate on refining the time series construction method, enhancing data consistency, and improving the accuracy and reliability of research results. These suggested improvements will contribute to a more thorough understanding of the dynamic patterns in ecological quality.

## Conclusions

This research utilizes Landsat series data from 2000 to 2020 in Yuxi, employing the GEE platform and HANTS to optimize the data. It establishes a long time series of RSEI based on four ecological indices during the vegetation growing season, aiming to evaluate ecological quality and analyze spatiotemporal patterns. The key findings of the study are: (1) Vegetation growing season data is essential for accurate RSEI assessment. (2) HANTS effectively fills missing data in the Landsat series, thereby improving the quality of ecological index data. (3) The overall evaluation of RSEI for Yuxi is moderate, displaying a center-edge pattern with better quality observed in the western and mountainous regions. (4) Over the 20-year period, RSEI fluctuates with a slight upward trend. Plains and river valleys showed deterioration, while mountainous areas showed improvement, mainly due to land-use changes such as deforestation and urbanization.

## Data Availability

The data sets used in the current study are available from the corresponding author on reasonable request.
